# Breast screening participation and retention among immigrants and nonimmigrants in British Columbia: A population‐based study

**DOI:** 10.1002/cam4.1608

**Published:** 2018-07-09

**Authors:** Ryan R. Woods, Kimberlyn M. McGrail, Erich V. Kliewer, Arminee Kazanjian, Colin Mar, Lisa Kan, Janette Sam, John J. Spinelli

**Affiliations:** ^1^ School of Population and Public Health University of British Columbia Vancouver BC Canada; ^2^ Population Oncology BC Cancer Vancouver BC Canada; ^3^ Community Health Sciences University of Manitoba Winnipeg MB Canada

**Keywords:** breast cancer, cancer screening, equity, immigrants, mammography, primary care

## Abstract

Breast cancer screening programs operate across Canada providing mammography to women in target age groups with the goal of reducing breast cancer mortality through early detection of tumors. Disparities in breast screening participation among socio‐demographic groups, including immigrants, have been reported in Canada. Our objectives were to: (1) assess breast screening participation and retention among immigrant and nonimmigrant women in British Columbia (BC), Canada; and (2) to characterize factors associated with screening among screening‐age recent immigrant women in BC. We examined 2 population‐based cohorts of women eligible for breast screening participation (537 783 women) and retention (281 052 women) using linked health and immigration data. Breast screening rates were presented according to socio‐demographic and health‐related variables stratified by birth country. Factors associated with screening among recent immigrant women were explored using Poisson regression. We observed marked variation in screening participation across birth country cohorts. Eastern European/Central Asian women showed low participation (37.9%) with rates from individual countries ranging from 35.0% to 49.0%. Participation rates for immigrant women from the most common birth countries, such as China/Macau/Hong Kong/Taiwan (45.7%), India (44.5%), the Philippines (45.9%), and South Korea (39.0%), were lower than the nonimmigrant rates (51.2%). Retention rates showed less variation by birth country; however, some disparities between immigrant and nonimmigrant groups persisted. Associations between screening indicators and study factors varied considerably across immigrant groups. Primary care physician visits were consistently positively associated with screening participation; this variable was also the only predictor associated with screening within each of the groups of recent immigrants. Our study provides unique data on both screening participation and retention among Canadian immigrant women compiled by individual country of birth. Our results are further demonstration that screening disparities exist among immigrant populations as well as in comparison with nonimmigrant women.

## 1 INTRODUCTION

Programmatic breast cancer screening with mammography is offered across Canada in an effort to detect tumors at earlier stages and reduce mortality from breast cancer. Screening mammograms are publicly funded in all Canadian provinces for women in target age groups yet in most Canadian jurisdictions participation rates remain well below the national target level of 70%.^1^ Breast screening participation rates are a composite measure of a program's ability to attract the target population to screen and their ability to retain this population throughout the duration of their screening eligibility. Recent screening retention rates in Canada have been similarly disappointing with some jurisdictions reporting declining retention^2^, ^3^ and 30‐month retention rates of first time participants below 50%.^2^

Disappointing breast screening participation rates as well as a desire to assess potential health inequities in cancer screening have motivated several recent investigations into potential screening disparities among socio‐demographic groups in Canada.^4^, ^5^, ^6^, ^7^, ^8^, ^9^, ^10^, ^11^, ^12^ Studies that have specifically examined breast screening have found screening disparities among immigrant subpopulations, as well as associations between mammography rates and duration of residence in Canada, primary care physician characteristics, primary care contacts, and other health and socio‐demographic variables.^8^, ^9^, ^10^, ^11^, ^12^ Research in Ontario, Canada identified disparities in screening rates among immigrant groups defined by world region of birth, including that South Asians had the lowest breast screening rates among all groups examined.^9^ Further research from the same population suggests that South Asian women may have more advanced breast tumors at the time of diagnosis.^13^, ^14^ Differences in screening rates within the immigrant population by length of residence in Canada have been reported, with much lower participation among more recent immigrants.^10^, ^12^

The world region categorization used in prior Canadian studies^8^, ^9^, ^11^ pooled women from a diverse set of countries, all of which have sizeable populations in Canada. For example, the East Asia/Pacific group included Filipino, Chinese, Korean, and Japanese immigrants. These groups may face different barriers to breast screening and have different screening patterns, but these potential differences are masked when data are examined only by world region of birth. Further, the composition of immigrant populations within each of the world region groups in terms of country of birth may differ across Canadian regions. For example, in Ontario, data from the 2016 Canadian census identified that 22% of the South Asian population originated from Pakistan while 55% were born in India.^15^ This same census identified the percentage of South Asian immigrants living in BC hailing from Pakistan and India as 6% and 90%, respectively. Thus interpreting regional differences in immigrant breast screening rates may be facilitated by examining rates by individual birth country. Prior breast screening studies generally focused on screening participation as the primary endpoint. Screening retention—defined simply as repeat screening according to guidelines—is an important performance indicator for cancer screening programs and may also show disparities among immigrant populations.

British Columbia has several strengths as a population within which to examine cancer screening patterns among Canadian immigrants. First, the 2016 Canadian Census showed that more than 1.29 million individuals, or 28.3% of the province's population, are foreign‐born.^15^ Second, BC's population is culturally diverse, with immigration data demonstrating a large number of recent Asian immigrants with the most common source countries being the Philippines, China, India, and the Republic of Korea.^16^ According to the 2016 Census, BC's total immigrant population includes significant numbers of immigrants from Asia (>750 000), Europe (>300 000), the Americas (>110 000), Africa (>40 000), and Oceania (>30 000).

The objectives of our study were: (1) to assess both screening participation and retention rates among BC's most common immigrant sub‐populations defined by country of birth; (2) to compare screening rates in these populations to those of nonimmigrant women; (3) to assess how breast screening rates vary with socio‐demographic and health‐related variables within these populations; and (4) to offer a specific focus on screening‐eligible recent immigrant (<10 years in Canada) women in terms of personal and health‐related characteristics and the associations between these factors and breast screening.

## 2 METHODS

### 2.1 Data sources and study setting

This study utilized several population‐based administrative databases from health and other government agencies via a comprehensive research data application facilitated through Population Data BC. Approval from all data stewards was obtained prior to data access. Specific details regarding the data sources accessed are provided in Table 1 and include: a provincial central demographics file, vital statistics death data, provincial cancer registry diagnoses, breast screening program data, fee‐for‐service physician payment information, in‐patient hospitalization and day surgery information, and federal immigration information.

**Table 1:** Details of data sources accessed for the present study

Database	Description	Years of data utilized by present study
Screening Mammography Program of British Columbia (SMPBC) database^38^	Includes information on SMPBC clients including demographics, self‐reported breast cancer risk factors, screening mammogram information, and results. The SMPBC database has captured information on clients since the program's inception in 1988.	1988‐2014
BC Cancer Registry (BCCR)^39^	A population‐based registry of all cases of cancer diagnosed in BC residents since 1970. Data from BCCR can be linked to other data sources from 1985 on as this was the first year that the provincial personal health number was consistently captured across health databases in BC.	1985‐2014
Medical Services Plan (MSP) physician payment file^40^	Includes all services provided by fee‐for‐service practitioners to individuals and billed to BC's Medical Services Plan. MSP is BC's public universal health coverage plan. Data include service dates, fee codes, and diagnoses responsible for paid physician services.	2008‐2014
Consolidation file^41^	The central demographic file containing residential and health coverage information for all individuals registered with MSP or who receive health services in BC	2008‐2014
Discharge Abstract Database (DAD)^42^	Includes data on hospital discharges, transfers, and deaths of in‐patients as well as day surgery admissions to BC acute care facilities. This data set includes patient and facility information as well as clinical details (including in‐hospital interventions) associated with the patient's hospital stay.	1985‐2014
Citizenship and Immigration Canada database^43^	Includes immigration details on permanent residents who immigrated to Canada between 1985 and 2012. Information includes details on countries of birth, last residence and citizenship, immigrant class, year of arrival and landing as well as socioeconomic information such as education‐level, occupation skills, and Canadian language proficiency. Limited to those immigrants who at one point were registered in BC's health coverage plan and thus were identified in the Consolidation file described above.	1985‐2012
BC Vital Statistics Agency database^44^	Captures all deaths registered in BC.	2010‐2014

British Columbia, Canada has a universal, publicly funded healthcare system that fully funds breast cancer screening mammograms through a provincial screening program. Current provincial guidelines recommend that average risk women age 50‐74 receive a mammogram every 2 years.^17^ The Canadian Task Force on Preventive Health Care recommendation on breast screening also recommended women in this age group screen with mammography every 2‐3 years.^18^ The single‐payer health system in British Columbia means that the data sets utilized for this study generally capture health transactions for all residents of the province who are registered in the government‐funded health system.

Research ethics approval was obtained from the University of British Columbia—BC Cancer Agency Research Ethics Board. The identities of all individuals in study data sets were replaced with study‐specific random numbers that permitted linkage across the various data sources while protecting confidentiality of all individuals.

### 2.2 Cohort derivation

#### 2.2.1 Participation cohort

The study cohort to examine screening participation was identified from the provincial health registration file and consisted of all women in BC who were aged 50‐69 years for the entire period from 1 January 2013 to 31 December 2014. This age group was chosen to align both with prior studies of breast screening participation and to reflect an age group within which average risk women have generally been recommended to screen biennially in Canada. Women were excluded if they had a diagnosis of breast cancer or mastectomy prior to 1 January 2013 were not continuously registered in the provincial health insurance plan 1 from January 2011 through the study period, or died prior to 31 December 2014. Women had to be registered over this entire period in order to characterize health‐service use and other health measures over a 2‐year look‐back period (2011‐2012) prior to the interval over which we calculated our study outcome (2013‐2014).

#### 2.2.2 Retention rate cohort

For the retention rate outcome, we examined a cohort of all screening eligible women who received a screening mammogram (the “index” screen) through the provincial screening mammography program of BC (SMPBC) between 1 January 2010 and 30 June 2012. These dates were chosen to permit a minimum of 30 months of follow‐up on each cohort member in order to determine a 30‐month retention rate. A 30‐month retention rate endpoint was chosen to align with the definition reported as a performance indicator by breast screening programs in Canada.^1^ As with the participation cohort, women were considered eligible if they were between 50 and 69 years of age for the entire period from the date of the index mammogram to the end of follow‐up (30‐months after their index mammogram). We further restricted this group to those who maintained provincial health coverage for the 2‐year period prior to the index mammogram to permit the evaluation of health service use for cohort members. Women were excluded if they died, developed breast cancer, had a mastectomy or discontinued provincial health coverage prior to the date of their next screen or the end of follow‐up. In the event women had 2 mammograms in the study period, we chose the first mammogram as the index mammogram.

### 2.3 Study outcomes and variable definitions

The primary study endpoints were the screening participation rate and 30‐month screening retention rate. The participation rate was defined as the number of women having a screening mammogram performed through the SMPBC between 1 January 2013 and 31 December 2014 out of the number of eligible women in the cohort. The retention rate was calculated as the number of women who had a screening mammogram performed through the SMPBC within 30 months of their index mammogram out of the total number of women who were eligible to be re‐screened over that period (ie, the number of women in the retention rate cohort). Diagnostic mammograms are not performed through the SMPBC in BC and are billed directly to the provincial health system by radiologists and can be booked only with a referral from a physician. However, it is unknown the extent to which women utilize diagnostic mammograms in the province for screening purposes. Thus, as a sensitivity analysis of the participation rate, we further included any bilateral mammograms billed directly to the health system (henceforth termed “diagnostic” mammograms) in the study period for any women who did not have a screening mammogram performed within the SMPBC.

We created study groups of nonimmigrant and immigrant women through linkage of the study cohorts to the immigration data. Any cohort member that did not link to the immigration data was assumed to be a nonimmigrant woman. Available immigration data included only individuals who immigrated to Canada between 1985 and 2012 and thus women who immigrated prior to 1985 cannot be distinguished in our data from nonimmigrant women. Our main analyses aimed to present screening rates by birth country as identified in the immigration file. Geopolitical changes that have taken place over the immigration dates covered by this data file necessitated combining some countries into single groups: countries of the former Union of Socialist Soviet Republics (USSR) were assembled into a “Former USSR State” group; countries of the former Yugoslavia were aggregated into “Former Yugoslavia”; women from the People's Republic of China, Macau, Hong Kong, and Taiwan were combined into a single group labeled “CMHT” in all tables and figures. We created world regions based primarily on groupings of countries used by the World Bank^19^ and consistent with other recent Canadian studies.^9^, ^20^ Immigrant women from countries with <100 total women were pooled into an “Other Immigrant” group within each world region.

Several socio‐demographic and health‐related measures were generated from the data sources identified in Table 1 in order to characterize study cohorts and examine correlates of breast screening. These variables included age, income quintile, rural residence, prior breast screening, index mammogram result, breast cancer family history, primary care physician (PCP) visits, the number of Johns Hopkins major aggregate diagnosis groups (ADGs),^21^ duration of residence in Canada, immigration class and application type, as well as Canadian language proficiency and education level at the time of landing. The full definitions of these variables can be found in Table 2.

**Table 2:** Definitions of study variables

Variable	Relevant cohort and population	Definition
Age	Participation and Retention; all women	In years; calculated from date of birth to the start of cohort follow‐up. Categorized into 2 groups: 50‐59 and 60‐69 years
Income quintile	Participation and Retention; all women	Derived from postal code of residence at the start of follow‐up and categorized into 5 quintiles. This is based on information captured in the 2006 Canadian census and compiled by residential postal codes
Rural residence	Participation and Retention; all women	Derived from postal code of residence at the start of follow‐up. Postal codes associated with communities with populations of <10 000 were assigned to rural; community sizes of ≥10 000 were assigned to urban
Prior breast screening	Participation and Retention; all women	The presence of any mammogram performed by the SMPBC prior to the start of follow‐up was taken to mean a prior history of screening; women with no documented SMPBC mammogram were assumed to have no prior screening history
Family history of breast cancer	Retention; all women	Based on self‐reported breast cancer history on the SMPBC client questionnaire. Women could indicate presence or absence of family history; women who did not complete this question were coded as unknown
Index screen results	Retention; all women	Based on index mammogram result identified in SMPBC database. Categorized as normal or abnormal result
Primary care physician visits	Participation and Retention; all women	The number of primary care physician office visits identified from the physician payment file within a 2‐year look‐back window prior to the start of follow‐up. Categorized into: 0, 1‐4, 5‐9, 10‐14, 15+
Number of major ADGs	Participation and Retention; all women	Based on the Johns Hopkins ACG/ADG system. The number of major ADGs identified was categorized into 0, 1, 2, or ≥3
Duration of residence in Canada	Participation and Retention; immigrants only	Calculated from date of landing in Canada identified in the immigration data to the start of cohort follow‐up. Categorized into 4 groups: <5, 5‐9, 10‐19, and ≥20 years
Canadian language proficiency	Participation; recent immigrants only	Based on the immigration data and reflects proficiency at the time of landing. Proficiency in either English or French is taken as having proficiency in Canadian language(s); no reported proficiency in either language taken as “none”
Education level	Participation; recent immigrants only	Based on the immigration data and reflects highest attained education at the time of landing
Immigration applicant type	Participation; recent immigrants only	Based on the immigration data and coded to principal, dependent, or other applicant type
Immigration class	Participation; recent immigrants only	Based on the immigration data and coded to economic, family, refugee, or other class

ADG, aggregate diagnosis groups; SMPBC, Screening Mammography Program of BC.

### 2.4 Statistical analysis

Age‐standardized participation and retention rates were calculated according to country and world region of birth using the age‐distribution of the nonimmigrant women as the standard population. Rates were generated for all countries with a minimum of 100 eligible women and presented graphically with 95% confidence intervals.

For birth countries for which there were at least 2000 women in the participation cohort, we undertook further analyses examining both the characteristics of the cohorts and their screening endpoints. This minimum sample size was chosen in order to obtain reasonable confidence interval widths for participation rates. Socio‐demographic and health measures were compared across immigrant groups and nonimmigrant populations using descriptive statistics. Screening participation and retention rates were generated by study group, both overall and stratified by other key variables, to explore the variation in screening endpoints; exact 95% confidence intervals were calculated for both endpoints.

We further examined the characteristics of screening eligible immigrants who had resided in Canada for <10 years (“recent” immigrants). Descriptive statistics for socio‐demographic, healthcare and immigration factors were generated by country of birth for women from the 8 most common birth countries. These countries were chosen as they accounted for more than 80% of the recent immigrant population, and each had a sufficient sample size to calculate participation rates. Recent immigrant women from countries other than these 8 were pooled into an “Other Immigrant” category for this analysis. We calculated participation rates according to these same variables within each birth country cohort. To identify independent predictors of screening for each birth country cohort, we used Poisson regression models with adjustment to the parameter estimate variances to permit a direct estimation of the adjusted relative risks as the endpoint of interest (screening participation) was not rare.^22^ Separate models were fit for each of the immigrant groups allowing for different variables to be selected or different effect estimates within each group. Categorical variables with <10 women within one of the categories were grouped with adjacent categories; binary explanatory variables with <10 women in one of the categories were not considered for that specific immigrant group. These decisions were made to avoid difficulties with model convergence. Terms were considered in an initial model containing all predictor variables with a sequence of generalized score tests^23^ used to backward eliminate variables not significantly associated with participation.

All analyses were conducted using the Statistical Analysis Software (SAS) version 9.4 (SAS Institute, Cary, NC, USA) and the R statistical computing software version 3.3.2 (http://www.cran.r-project.org/).

## 3 RESULTS

### 3.1 Breast screening participation

The participation cohort included 537 783 women of whom 85 902 (16.0%) were identified as immigrants. The majority of the immigrant population who were eligible for breast screening during our study period hailed from Asia with more than 59.0% of the immigrant population in our cohort born in CMHT, the Philippines or India (Table 3). Among immigrant groups, duration of residence in Canada was highly variable. For example, the majority of Vietnamese immigrants (63.7%) had resided in Canada for 20 or more years; this contrasted with the Indian immigrant group where only 12.6% had been in Canada 20 years or more. Compared to nonimmigrant women, the immigrant sub‐populations were younger with a higher frequency of women in the 50‐59 age group; immigrant Indian women, however, were more commonly aged 60‐69 (52.6%), higher than other study groups (21.2%‐40.4%). Most of the cohort resided in urban areas, with immigrant populations generally showing a higher frequency of urban residence. Korean and Chinese immigrant women showed a much higher frequency (~15%) of women who had not seen a physician in the 2‐year look‐back period.

**Table 3:** Characteristics of screening participation cohort

Variable	Subgroup	Non‐immigrant (N = 451 881)	CMHT (N = 30 185)	Philippines (N = 10 911)	India (N = 9958)	South Korea (N = 4028)	Iran (N = 3517)	United Kingdom (N = 2692)	United States (N = 2572)	Vietnam (N = 2089)	Other Immigrants (N = 19 950)
Age	50‐59	269 217 (59.6%)	21 637 (71.7%)	8597 (78.8%)	4721 (47.4%)	3075 (76.3%)	2507 (71.3%)	2020 (75.0%)	1713 (66.6%)	1579 (75.6%)	14 681 (73.6%)
	60‐69	182 664 (40.4%)	8548 (28.3%)	2314 (21.2%)	5237 (52.6%)	953 (23.7%)	1010 (28.7%)	672 (25.0%)	859 (33.4%)	510 (24.4%)	5269 (26.4%)
Urban/Rural residence	Urban	377 369 (83.5%)	30 091 (99.7%)	10 675 (97.8%)	9790 (98.3%)	3953 (98.1%)	3503 (99.6%)	2315 (86.0%)	1974 (76.7%)	2083 (99.7%)	18 808 (94.3%)
	Unknown	121 (0.0%)	6 (0.0%)	<5 (0.0%)	<5 (0.0%)	<5 (0.0%)	<5 (0.0%)	<5 (0.0%)	<5 (0.0%)	<5 (0.0%)	<5 (0.0%)
Income quintile	1 (lowest)	77 218 (17.1%)	8243 (27.3%)	3529 (32.3%)	2623 (26.3%)	708 (17.6%)	472 (13.4%)	301 (11.2%)	379 (14.7%)	716 (34.3%)	4787 (24.0%)
	2	82 661 (18.3%)	6649 (22.0%)	3107 (28.5%)	3489 (35.0%)	720 (17.9%)	706 (20.1%)	353 (13.1%)	421 (16.4%)	640 (30.6%)	4246 (21.3%)
	3	89 586 (19.8%)	5971 (19.8%)	2121 (19.4%)	2056 (20.6%)	826 (20.5%)	489 (13.9%)	501 (18.6%)	462 (18.0%)	386 (18.5%)	3921 (19.7%)
	4	97 307 (21.5%)	4606 (15.3%)	1304 (12.0%)	1050 (10.5%)	867 (21.5%)	729 (20.7%)	651 (24.2%)	510 (19.8%)	224 (10.7%)	3476 (17.4%)
	5 (highest)	101 004 (22.4%)	4434 (14.7%)	783 (7.2%)	732 (7.4%)	840 (20.9%)	999 (28.4%)	868 (32.2%)	767 (29.8%)	114 (5.5%)	3374 (16.9%)
	Unknown	4105 (0.9%)	282 (0.9%)	67 (0.6%)	8 (0.1%)	67 (1.7%)	122 (3.5%)	18 (0.7%)	33 (1.3%)	9 (0.4%)	146 (0.7%)
# Major ADG's	0	264 682 (58.6%)	21 459 (71.1%)	7279 (66.7%)	5577 (56.0%)	2851 (70.8%)	2116 (60.2%)	1722 (64.0%)	1585 (61.6%)	1337 (64.0%)	12 514 (62.7%)
	1	114 594 (25.4%)	5695 (18.9%)	2394 (21.9%)	2748 (27.6%)	772 (19.2%)	863 (24.5%)	650 (24.1%)	610 (23.7%)	489 (23.4%)	4761 (23.9%)
	2	40 356 (8.9%)	1604 (5.3%)	671 (6.1%)	972 (9.8%)	208 (5.2%)	315 (9.0%)	175 (6.5%)	212 (8.2%)	141 (6.7%)	1505 (7.5%)
	3+	17 945 (4.0%)	482 (1.6%)	221 (2.0%)	358 (3.6%)	52 (1.3%)	109 (3.1%)	60 (2.2%)	72 (2.8%)	47 (2.2%)	576 (2.9%)
	Unknown	14 304 (3.2%)	945 (3.1%)	346 (3.2%)	303 (3.0%)	145 (3.6%)	114 (3.2%)	85 (3.2%)	93 (3.6%)	75 (3.6%)	594 (3.0%)
# PCP visits	Median [IQR]	8.0 [4.0‐14.0]	7.0 [3.0‐13.0]	9.0 [5.0‐14.0]	14.0 [8.0‐21.0]	7.0 [2.0‐12.0]	12.0 [6.0‐18.0]	7.0 [4.0‐12.0]	7.0 [4.0‐12.0]	10.0 [5.0‐16.0]	8.0 [4.0‐14.0]
	0	24 489 (5.4%)	4428 (14.7%)	578 (5.3%)	279 (2.8%)	613 (15.2%)	256 (7.3%)	174 (6.5%)	176 (6.8%)	128 (6.1%)	1404 (7.0%)
	1‐4	94 031 (20.8%)	6285 (20.8%)	2029 (18.6%)	837 (8.4%)	939 (23.3%)	397 (11.3%)	640 (23.8%)	641 (24.9%)	313 (15.0%)	4022 (20.2%)
	5‐9	135 389 (30.0%)	8050 (26.7%)	3351 (30.7%)	1910 (19.2%)	1109 (27.5%)	779 (22.1%)	861 (32.0%)	806 (31.3%)	531 (25.4%)	5545 (27.8%)
	10‐14	92 818 (20.5%)	5717 (18.9%)	2561 (23.5%)	2233 (22.4%)	728 (18.1%)	772 (22.0%)	524 (19.5%)	488 (19.0%)	474 (22.7%)	4053 (20.3%)
	15+	105 154 (23.3%)	5705 (18.9%)	2392 (21.9%)	4699 (47.2%)	639 (15.9%)	1313 (37.3%)	493 (18.3%)	461 (17.9%)	643 (30.8%)	4926 (24.7%)
Prior screening	Yes	339 836 (75.2%)	21 581 (71.5%)	7346 (67.3%)	5898 (59.2%)	2743 (68.1%)	2725 (77.5%)	2017 (74.9%)	1758 (68.4%)	1491 (71.4%)	13 510 (67.7%)
Years of residence in Canada	Median [IQR]	NA	16.7 [12.7‐19.7]	17.8 [11.6‐21.0]	11.5 [6.8‐16.8]	13.8 [10.0‐18.2]	14.4 [9.5‐18.8]	19.5 [11.3‐23.6]	17.4 [7.5‐23.2]	21.5 [18.6‐24.3]	18.3 [12.3‐22.7]
	<5		1416 (4.7%)	930 (8.5%)	1538 (15.4%)	244 (6.1%)	295 (8.4%)	218 (8.1%)	290 (11.3%)	54 (2.6%)	1092 (5.5%)
	5‐9		3605 (11.9%)	1399 (12.8%)	2534 (25.4%)	762 (18.9%)	635 (18.1%)	381 (14.2%)	559 (21.7%)	137 (6.6%)	2406 (12.1%)
	10‐19		18 119 (60.0%)	4981 (45.7%)	4636 (46.6%)	2347 (58.3%)	1825 (51.9%)	811 (30.1%)	723 (28.1%)	567 (27.1%)	8348 (41.8%)
	20+		7045 (23.3%)	3601 (33.0%)	1250 (12.6%)	675 (16.8%)	762 (21.7%)	1282 (47.6%)	1000 (38.9%)	1331 (63.7%)	8104 (40.6%)

ADG, aggregate diagnosis group; CMHT, China, Macau, Hong Kong, Taiwan; IQR, inter‐quartile range; NA, not applicable; PCP, primary care physician.

**Figure 1:**
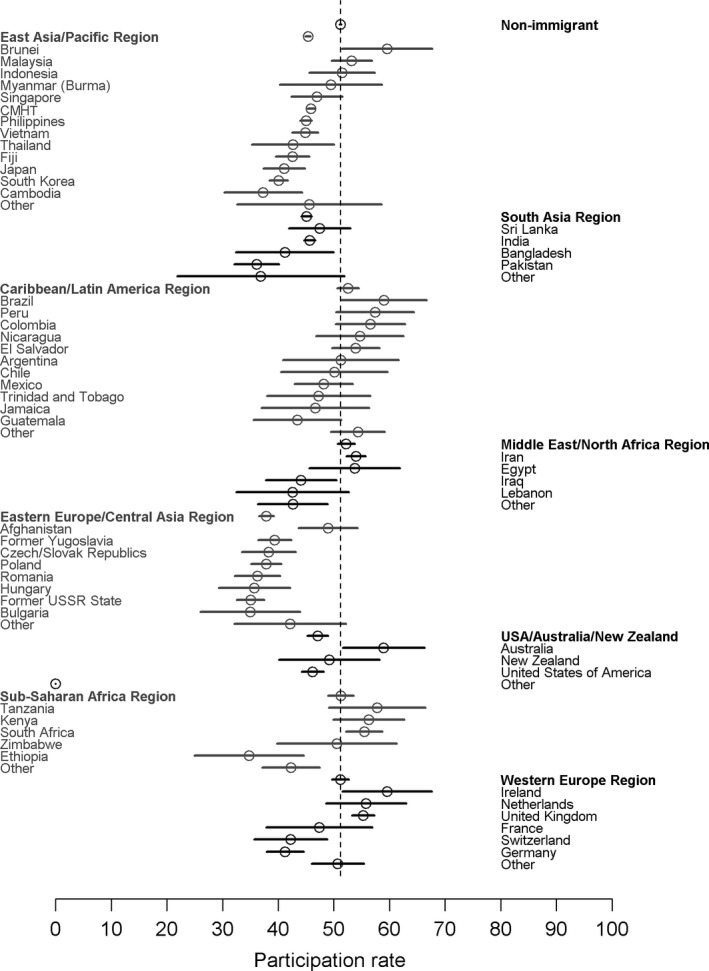
Age‐standardized screening participation rates by country of birth for countries with 100 or more women in the participation cohort. Vertical dashed line represents the nonimmigrant participation rate. CMHT, China, Macau, Hong Kong, and Taiwan

The age‐adjusted participation rates varied considerably by country and world region of birth (Figure 1). Women from the East Asia/Pacific region generally showed lower screening rates than the nonimmigrant population; however, women from some countries, particularly South‐East Asian countries (eg, Brunei, Malaysia, Indonesia) demonstrated similar rates to nonimmigrants. South Asian women also had lower participation rates than the nonimmigrant population; participation was particularly low for immigrants from Pakistan within this region. With the exception of women born in Afghanistan, Central Asian/Eastern European immigrants showed consistently lower participation (35.0%‐49.0%), with some of the lowest rates among the countries examined in our analysis. Age‐standardized participation rates and confidence intervals for all countries examined can be found in Table S1.

Table 4 shows the overall participation rates for the entire population as well as for birth countries with at least 2000 women in the cohort. The unadjusted participation rate for the entire cohort was 50.3%. Within this group of countries, there was large variation in the participation rates with South Korean women reporting the lowest participation (39.0%) and women from Iran (53.9%) the highest. Screening rates did not vary consistently with age across the immigrant populations. Among immigrant women aged 50‐59, participation was lowest for immigrants from South Korea (37.6%) and highest for those from the UK (54.3%). There was similar variation in participation among the immigrant women aged 60‐69 with the lowest rates in Indian women (41.1%) and highest in immigrants from the UK (57.0%). Participation in the nonimmigrant population was higher in the 60‐69 age group (55.1% vs 48.5% in the 50‐59 age group). These patterns resulted in some age‐specific disparities such as those observed for Indian women, where in the 50‐59 age group, the participation rate was identical to the rate among nonimmigrant women but in the 60‐69 age group it was almost 13% lower.

**Table 4:** Breast screening participation rates for screening eligible women by study factors

Variable	Subgroup	Population (N = 537 783)	Non‐immigrant (N = 451 881)	CMHT (N = 30 185)	Philippines (N = 10 911)	India (N = 9958)	South Korea (N = 4028)	Iran (N = 3517)	United Kingdom (N = 2692)	United States (N = 2572)	Vietnam (N = 2089)	Other Immigrants (N = 19 950)
All Women	All Women	50.3 [50.2, 50.4]	51.2 [51.0, 51.3]	45.7 [45.2, 46.3]	45.9 [44.9, 46.8]	44.5 [43.5, 45.5]	39.0 [37.5, 40.5]	53.9 [52.2, 55.5]	54.9 [53.0, 56.8]	46.1 [44.2, 48.1]	46.5 [44.3, 48.6]	44.5 [43.8, 45.2]
Age	50‐59	48.0 [47.8, 48.2]	48.5 [48.3, 48.7]	45.3 [44.6, 46.0]	46.8 [45.7, 47.8]	48.3 [46.8, 49.7]	37.6 [35.8, 39.3]	53.8 [51.8, 55.8]	54.3 [52.1, 56.4]	45.6 [43.2, 48.0]	48.1 [45.6, 50.6]	44.2 [43.4, 45.0]
	60‐69	53.9 [53.7, 54.2]	55.1 [54.9, 55.4]	46.8 [45.8, 47.9]	42.6 [40.6, 44.7]	41.1 [39.8, 42.5]	43.7 [40.5, 46.9]	54.0 [50.8, 57.1]	57.0 [53.2, 60.8]	47.1 [43.8, 50.5]	41.6 [37.3, 46.0]	45.5 [44.1, 46.8]
Urban/Rural residence	Urban	51.2 [51.0, 51.3]	52.3 [52.2, 52.5]	45.8 [45.2, 46.3]	46.0 [45.1, 47.0]	44.7 [43.7, 45.7]	38.9 [37.4, 40.4]	53.8 [52.1, 55.5]	55.7 [53.6, 57.7]	48.5 [46.3, 50.7]	46.4 [44.2, 48.5]	44.7 [44.0, 45.4]
	Rural	45.2 [44.8, 45.5]	45.3 [45.0, 45.7]	27.3 [18.3, 37.8]	39.0 [32.7, 45.5]	36.0 [28.6, 43.8]	44.6 [33.0, 56.6]	69.2 [38.6, 90.9]	50.4 [45.2, 55.6]	38.3 [34.4, 42.3]	80.0 [28.4, 99.5]	41.4 [38.6, 44.4]
Income quintile	1 (lowest)	43.0 [42.7, 43.3]	42.9 [42.5, 43.2]	44.5 [43.5, 45.6]	43.2 [41.5, 44.8]	43.5 [41.6, 45.4]	34.6 [31.1, 38.2]	50.4 [45.8, 55.0]	49.2 [43.4, 55.0]	42.2 [37.2, 47.4]	41.5 [37.8, 45.2]	41.4 [40.0, 42.8]
	2	48.3 [48.0, 48.6]	48.8 [48.5, 49.2]	47.8 [46.6, 49.1]	45.9 [44.1, 47.7]	44.7 [43.0, 46.4]	42.4 [38.7, 46.1]	52.1 [48.4, 55.9]	47.0 [41.7, 52.4]	43.7 [38.9, 48.6]	50.9 [47.0, 54.9]	43.7 [42.2, 45.2]
	3	51.1 [50.8, 51.4]	51.9 [51.6, 52.2]	47.4 [46.1, 48.7]	48.6 [46.5, 50.8]	45.5 [43.4, 47.7]	43.2 [39.8, 46.7]	57.1 [52.5, 61.5]	55.3 [50.8, 59.7]	44.6 [40.0, 49.3]	46.4 [41.3, 51.5]	45.1 [43.6, 46.7]
	4	52.7 [52.4, 53.0]	53.5 [53.2, 53.8]	45.0 [43.6, 46.5]	48.8 [46.0, 51.5]	43.0 [39.9, 46.0]	39.6 [36.3, 42.9]	53.9 [50.2, 57.6]	55.6 [51.7, 59.5]	47.3 [42.9, 51.7]	48.2 [41.5, 55.0]	46.3 [44.6, 47.9]
	5 (highest)	55.7 [55.4, 56.0]	56.8 [56.5, 57.1]	43.4 [41.9, 44.9]	46.1 [42.6, 49.7]	46.9 [43.2, 50.5]	36.5 [33.3, 39.9]	56.0 [52.8, 59.1]	59.9 [56.6, 63.2]	49.7 [46.1, 53.3]	49.1 [39.6, 58.7]	48.0 [46.3, 49.7]
# Major ADG's	0	48.3 [48.1, 48.4]	49.6 [49.4, 49.8]	41.1 [40.4, 41.8]	43.3 [42.1, 44.4]	41.2 [39.9, 42.5]	34.0 [32.2, 35.8]	49.4 [47.2, 51.5]	53.8 [51.4, 56.2]	44.0 [41.6, 46.5]	43.4 [40.7, 46.1]	41.7 [40.9, 42.6]
	1	54.4 [54.1, 54.6]	54.6 [54.3, 54.9]	57.6 [56.3, 58.9]	51.4 [49.4, 53.4]	48.0 [46.1, 49.8]	51.2 [47.6, 54.7]	60.4 [57.0, 63.7]	58.0 [54.1, 61.8]	50.3 [46.3, 54.4]	55.0 [50.5, 59.5]	49.2 [47.7, 50.6]
	2	53.6 [53.1, 54.0]	53.3 [52.9, 53.8]	60.3 [57.8, 62.7]	52.3 [48.5, 56.1]	51.2 [48.0, 54.4]	58.7 [51.6, 65.4]	59.4 [53.7, 64.8]	53.7 [46.0, 61.3]	50.0 [43.1, 56.9]	52.5 [43.9, 60.9]	53.0 [50.4, 55.5]
	3+	47.8 [47.2, 48.5]	47.2 [46.5, 47.9]	63.1 [58.6, 67.4]	54.3 [47.5, 61.0]	52.5 [47.2, 57.8]	53.8 [39.5, 67.8]	68.8 [59.2, 77.3]	56.7 [43.2, 69.4]	51.4 [39.3, 63.3]	31.9 [19.1, 47.1]	45.1 [41.0, 49.3]
	Unknown	50.7 [49.9, 51.4]	51.7 [50.9, 52.5]	45.7 [42.5, 49.0]	44.5 [39.2, 49.9]	43.9 [38.2, 49.7]	39.3 [31.3, 47.8]	57.9 [48.3, 67.1]	55.3 [44.1, 66.1]	40.9 [30.8, 51.5]	44.0 [32.5, 55.9]	44.3 [40.2, 48.4]
# PCP visits	0	14.3 [13.9, 14.7]	16.4 [16.0, 16.9]	5.6 [5.0, 6.4]	10.7 [8.3, 13.5]	8.6 [5.6, 12.5]	6.4 [4.6, 8.6]	9.4 [6.1, 13.6]	16.7 [11.5, 23.1]	13.1 [8.5, 19.0]	8.6 [4.4, 14.9]	12.2 [10.5, 14.0]
	1‐4	43.1 [42.8, 43.4]	44.6 [44.3, 44.9]	33.6 [32.4, 34.7]	33.8 [31.8, 35.9]	27.5 [24.5, 30.6]	30.0 [27.1, 33.1]	38.5 [33.7, 43.5]	45.5 [41.6, 49.4]	38.1 [34.3, 42.0]	33.2 [28.0, 38.7]	35.3 [33.8, 36.8]
	5‐9	54.2 [54.0, 54.5]	55.1 [54.9, 55.4]	51.5 [50.4, 52.6]	47.2 [45.5, 48.9]	40.4 [38.2, 42.7]	44.7 [41.8, 47.7]	52.8 [49.2, 56.3]	60.6 [57.3, 63.9]	52.4 [48.8, 55.9]	46.0 [41.7, 50.3]	46.4 [45.1, 47.7]
	10‐14	57.0 [56.7, 57.3]	57.5 [57.2, 57.8]	60.5 [59.2, 61.7]	51.9 [50.0, 53.9]	45.1 [43.0, 47.2]	50.4 [46.7, 54.1]	60.2 [56.7, 63.7]	65.6 [61.4, 69.7]	55.7 [51.2, 60.2]	49.2 [44.6, 53.8]	51.8 [50.2, 53.3]
	15+	55.0 [54.8, 55.3]	54.4 [54.1, 54.7]	67.3 [66.1, 68.5]	56.3 [54.3, 58.3]	51.1 [49.6, 52.5]	60.6 [56.7, 64.4]	64.1 [61.4, 66.7]	59.4 [55.0, 63.8]	48.8 [44.2, 53.5]	58.9 [55.0, 62.8]	53.3 [51.9, 54.7]
Prior screening	Yes	65.1 [65.0, 65.3]	65.7 [65.5, 65.8]	61.1 [60.4, 61.7]	63.3 [62.2, 64.4]	64.3 [63.0, 65.5]	53.4 [51.6, 55.3]	64.7 [62.8, 66.5]	69.5 [67.4, 71.5]	63.3 [61.0, 65.6]	61.4 [58.9, 63.9]	61.4 [60.6, 62.2]
Years of residence in Canada^a^	<5	37.0 [35.8, 38.2]	NA	33.5 [31.1, 36.1]	37.2 [34.1, 40.4]	34.9 [32.5, 37.3]	25.0 [19.7, 30.9]	54.9 [49.0, 60.7]	53.7 [46.8, 60.4]	41.7 [36.0, 47.6]	48.1 [34.3, 62.2]	36.9 [34.0, 39.8]
	5‐9	39.3 [38.5, 40.2]		35.8 [34.2, 37.3]	43.1 [40.5, 45.7]	38.6 [36.7, 40.5]	29.9 [26.7, 33.3]	55.0 [51.0, 58.9]	52.0 [46.8, 57.1]	42.4 [38.3, 46.6]	35.8 [27.8, 44.4]	39.6 [37.6, 41.6]
	10‐19	45.9 [45.4, 46.3]		45.4 [44.6, 46.1]	45.6 [44.2, 47.0]	48.7 [47.2, 50.1]	40.8 [38.8, 42.8]	53.6 [51.3, 56.0]	54.0 [50.5, 57.5]	47.6 [43.9, 51.3]	47.4 [43.3, 51.6]	44.2 [43.1, 45.3]
	20+	50.6 [50.0, 51.2]		54.2 [53.1, 55.4]	49.6 [47.9, 51.2]	53.0 [50.1, 55.8]	48.1 [44.3, 52.0]	53.0 [49.4, 56.6]	56.6 [53.9, 59.4]	48.4 [45.3, 51.5]	47.1 [44.4, 49.8]	47.4 [46.3, 48.5]

ADG, aggregate diagnosis group; CMHT, China, Macau, Hong Kong, Taiwan; PCP, primary care physician.

a Years of residence in Canada for the “Population” column refers to the pooled group of all immigrants.

Screening participation increased with income quintile in the nonimmigrant population; however, the relationship varied across immigrant populations (Table 4). Screening rates increased with PCP visits for almost all of the groups. Screening rates were very low (5.6%‐16.7%) for women who had no contact with a PCP in the 2 years prior to the start of follow‐up. Participation generally increased with duration of residence in Canada. This was most evident for women from CMHT, India, and South Korea where the absolute difference in participation between the most recent and longest‐term immigrants (≥20 years in Canada) approached or exceeded 20%. Screening rates for most long‐term immigrant groups approached those of nonimmigrant women. For completeness, and to enable comparisons with other prior Canadian studies, we have included in the supplemental materials (Table S2) a table of participation rates stratified by key study variables and the birth world region group used in prior Canadian studies.

The sensitivity analysis, where diagnostic mammograms performed outside the screening program were included in the participation endpoint, yielded nearly identical results to the primary analysis (data not shown). The overall participation rate for the cohort increased to 54.2% with the ordering of the various subpopulations remaining largely the same. Although the inclusion of these mammograms increased some groups’ participation rates approximately in‐line with the increase seen in the overall population rate (4%), the Iranian women's rate increased 8% with these additional mammograms. In contrast, the rate for Indian women increased only 1.8%. Generally, relationships between participation and other variables remained similar to the main analysis.

### 3.2 Breast screening retention

The retention rate cohort included 281 052 women of which 12.8% were identified as immigrants (Table 5). The age distribution of the retention rate cohort closely resembled that of the participation cohort for most groups. Indian immigrants tended to be younger in the retention cohort compared to the participation cohort. Although there was still notable variation in the number of physician contacts across study groups in the retention cohort, there were far fewer women in any study group with no PCP contacts (range 0.3%‐1.8% in the retention rate cohort vs 2.8%‐15.2% in the participation cohort). Indian women reported the lowest rate of prior screening such that the index mammogram used in our study was the first screen for 22.7% of the Indian group compared to only 5.6% in the nonimmigrant group.

**Table 5:** Characteristics of screening retention cohort

Variable	Subgroup	Non‐immigrant (N = 245 123)	CMHT (N = 12 863)	Philippines (N = 4324)	India (N = 4054)	Iran (N = 1716)	South Korea (N = 1553)	United Kingdom (N = 1318)	United States (N = 1141)	Vietnam (N = 849)	Other Immigrants (N = 8111)
Age	50‐59	146 034 (59.6%)	9505 (73.9%)	3566 (82.5%)	2190 (54.0%)	1289 (75.1%)	1148 (73.9%)	973 (73.8%)	797 (69.9%)	662 (78.0%)	6098 (75.2%)
	60‐69	99 089 (40.4%)	3358 (26.1%)	758 (17.5%)	1864 (46.0%)	427 (24.9%)	405 (26.1%)	345 (26.2%)	344 (30.1%)	187 (22.0%)	2013 (24.8%)
Urban/Rural residence	Urban	209 059 (85.3%)	12 844 (99.9%)	4239 (98.0%)	3998 (98.6%)	1707 (99.5%)	1520 (97.9%)	1127 (85.5%)	905 (79.3%)	848 (99.9%)	7689 (94.8%)
	Unknown	26 (0.0%)	0 (0.0%)	0 (0.0%)	<5 (0.0%)	0 (0.0%)	0 (0.0%)	0 (0.0%)	0 (0.0%)	0 (0.0%)	0 (0.0%)
Income quintile	1 (lowest)	35 448 (14.5%)	3441 (26.8%)	1364 (31.5%)	1147 (28.3%)	221 (12.9%)	274 (17.6%)	140 (10.6%)	152 (13.3%)	274 (32.3%)	1819 (22.4%)
	2	42 973 (17.5%)	2950 (22.9%)	1181 (27.3%)	1380 (34.0%)	343 (20.0%)	285 (18.4%)	173 (13.1%)	171 (15.0%)	284 (33.5%)	1760 (21.7%)
	3	48 975 (20.0%)	2685 (20.9%)	872 (20.2%)	816 (20.1%)	247 (14.4%)	318 (20.5%)	258 (19.6%)	209 (18.3%)	162 (19.1%)	1582 (19.5%)
	4	54 898 (22.4%)	1946 (15.1%)	550 (12.7%)	435 (10.7%)	369 (21.5%)	324 (20.9%)	304 (23.1%)	242 (21.2%)	82 (9.7%)	1441 (17.8%)
	5 (highest)	61 036 (24.9%)	1768 (13.7%)	333 (7.7%)	271 (6.7%)	500 (29.1%)	338 (21.8%)	437 (33.2%)	353 (30.9%)	44 (5.2%)	1458 (18.0%)
	Unknown	1793 (0.7%)	73 (0.6%)	24 (0.6%)	5 (0.1%)	36 (2.1%)	14 (0.9%)	6 (0.5%)	14 (1.2%)	<5 (<0.6%)	51 (0.6%)
# Major ADG's	0	132 370 (54.0%)	7467 (58.1%)	2497 (57.7%)	2080 (51.3%)	934 (54.4%)	863 (55.6%)	749 (56.8%)	597 (52.3%)	473 (55.7%)	4336 (53.5%)
	1	62 882 (25.7%)	2944 (22.9%)	1029 (23.8%)	1104 (27.2%)	429 (25.0%)	380 (24.5%)	332 (25.2%)	283 (24.8%)	207 (24.4%)	2078 (25.6%)
	2	21 292 (8.7%)	936 (7.3%)	312 (7.2%)	370 (9.1%)	154 (9.0%)	91 (5.9%)	95 (7.2%)	96 (8.4%)	67 (7.9%)	698 (8.6%)
	3+	8162 (3.3%)	323 (2.5%)	125 (2.9%)	152 (3.7%)	52 (3.0%)	36 (2.3%)	34 (2.6%)	34 (3.0%)	26 (3.1%)	219 (2.7%)
	Unknown	20 417 (8.3%)	1193 (9.3%)	361 (8.3%)	348 (8.6%)	147 (8.6%)	183 (11.8%)	108 (8.2%)	131 (11.5%)	76 (9.0%)	780 (9.6%)
# PCP visits	Median [IQR]	9.0 [5.0‐15.0]	10.0 [6.0‐16.0]	11.0 [6.0‐16.0]	15.0 [10.0‐23.0]	13.0 [9.0‐20.0]	9.0 [6.0‐14.0]	9.0 [5.0‐13.0]	8.0 [5.0‐13.0]	13.0 [8.0‐19.0]	10.0 [6.0‐16.0]
	0	3579 (1.5%)	177 (1.4%)	51 (1.2%)	14 (0.3%)	8 (0.5%)	27 (1.7%)	24 (1.8%)	17 (1.5%)	<5 (<0.6%)	120 (1.5%)
	1‐4	44 110 (18.0%)	1897 (14.7%)	576 (13.3%)	181 (4.5%)	138 (8.0%)	270 (17.4%)	261 (19.8%)	264 (23.1%)	77 (9.1%)	1289 (15.9%)
	5‐9	77 668 (31.7%)	3754 (29.2%)	1244 (28.8%)	726 (17.9%)	368 (21.4%)	504 (32.5%)	449 (34.1%)	391 (34.3%)	195 (23.0%)	2333 (28.8%)
	10‐14	56 323 (23.0%)	3195 (24.8%)	1172 (27.1%)	946 (23.3%)	419 (24.4%)	384 (24.7%)	300 (22.8%)	232 (20.3%)	211 (24.9%)	1777 (21.9%)
	15+	63 443 (25.9%)	3840 (29.9%)	1281 (29.6%)	2187 (53.9%)	783 (45.6%)	368 (23.7%)	284 (21.5%)	237 (20.8%)	363 (42.8%)	2592 (32.0%)
Family history of breast cancer	Yes	36 258 (14.8%)	1088 (8.5%)	390 (9.0%)	190 (4.7%)	148 (8.6%)	96 (6.2%)	156 (11.8%)	195 (17.1%)	53 (6.2%)	750 (9.2%)
	Unknown	16 359 (6.7%)	674 (5.2%)	272 (6.3%)	269 (6.6%)	90 (5.2%)	83 (5.3%)	80 (6.1%)	59 (5.2%)	66 (7.8%)	538 (6.6%)
Prior screening	Yes	231 474 (94.4%)	12 048 (93.7%)	3830 (88.6%)	3135 (77.3%)	1520 (88.6%)	1377 (88.7%)	1215 (92.2%)	1029 (90.2%)	768 (90.5%)	7242 (89.3%)
Index screen result	Abnormal	16 118 (6.6%)	646 (5.0%)	370 (8.6%)	384 (9.5%)	128 (7.5%)	68 (4.4%)	89 (6.8%)	90 (7.9%)	50 (5.9%)	627 (7.7%)
Years of residence in Canada	Median [IQR]	NA	15.8 [13.1‐18.7]	16.7 [11.6‐19.8]	11.8 [7.6‐16.0]	13.0 [8.6‐18.1]	13.9 [10.5‐17.9]	18.1 [11.1‐21.9]	16.2 [7.0‐21.8]	19.7 [16.6‐22.6]	17.4 [12.0‐21.3]
	<5		451 (3.5%)	367 (8.5%)	571 (14.1%)	180 (10.5%)	75 (4.8%)	121 (9.2%)	195 (17.1%)	31 (3.7%)	428 (5.3%)
	5‐9		1302 (10.1%)	516 (11.9%)	1049 (25.9%)	360 (21.0%)	271 (17.5%)	176 (13.4%)	185 (16.2%)	51 (6.0%)	1033 (12.7%)
	10‐19		8900 (69.2%)	2429 (56.2%)	2031 (50.1%)	908 (52.9%)	975 (62.8%)	520 (39.5%)	397 (34.8%)	369 (43.5%)	3926 (48.4%)
	20+		2210 (17.2%)	1012 (23.4%)	403 (9.9%)	268 (15.6%)	232 (14.9%)	501 (38.0%)	364 (31.9%)	398 (46.9%)	2724 (33.6%)

ADG, aggregate diagnosis group; CMHT, China, Macau, Hong Kong, Taiwan; IQR, inter‐quartile range; PCP, primary care physician.

**Figure 2:**
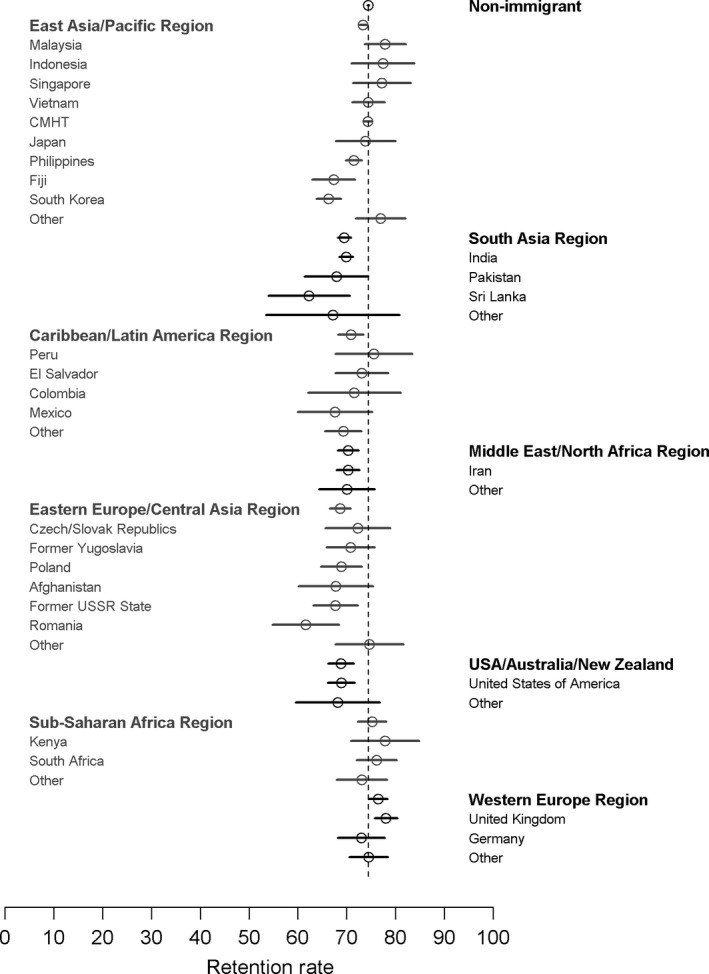
Age‐standardized 30‐month screening retention rates by country of birth for countries with 100 or more women in the retention cohort. Vertical dashed line represents the non‐immigrant retention rate. CMHT, China, Macau, Hong Kong, and Taiwan

Figure 2 shows the age‐standardized 30‐month retention by country of birth. There was much less variation in retention rates among birth countries than in the participation rates (Figure 1). For example, although the Eastern European/Central Asian immigrants still had numerically lower retention rates compared to nonimmigrants, these countries were much closer to the nonimmigrant rates (eg, rates within this region ranged from 61.6% to 74.6% vs 74.4% for nonimmigrant women). Some immigrant groups that had low participation within their world region group, such as Germany or Japan, demonstrated retention rates consistent with the nonimmigrant population or in the case of Pakistan similar to the regional rate. Because retention rates by country may be influenced by the fraction of index mammograms in each group that represented women's first time screening, we have provided this additional data in Table S3.

The overall 30‐month retention rate for the study cohort was 74.0% (44.4% for first time screeners vs 76.0% for those who had previously screened). Retention rates across immigrant groups ranged from 64.9% in Korean women to 77.4% for women from the United Kingdom (Table 6). Retention rates showed modest increases with age among nonimmigrant and South Korean women, whereas most other groups showed little association with age. Indian women who showed a decrease of approximately 7% in participation with age showed similar retention rates in both age groups (~70%). Among first‐time screeners, South Korean women had the lowest retention (40.3%), while immigrants from the UK (61.2%), Iran (52.0%), and the Philippines (52.6%) had the highest. Retention rates generally increased with greater physician contact, with most groups having the lowest retention rates among women who had no contact with a primary care physician. Among women who had at least 1 PCP contact in the look‐back period, the variation in retention rates across the levels of PCP visits differed by group. For example, among immigrants from the USA and UK, the range in observed retention rates across levels of PCP visits was no more than 4.8% and 7.4%, respectively; the range was significantly higher among women from Iran (13.8%), Vietnam (14.4%), and CMHT (16.9%).

**Table 6:** Thirty‐month screening retention rates for screening eligible women by study factors

Variable	Subgroup	Population (N = 281 052)	Non‐immigrant (N = 245 123)	CMHT (N = 12 863)	Philippines (N = 4324)	India (N = 4054)	Iran (N = 1716)	South Korea (N = 1553)	United Kingdom (N = 1318)	United States (N = 1141)	Vietnam (N = 849)	Other Immigrants (N = 8111)
All women	All women	74.0 [73.9, 74.2]	74.4 [74.2, 74.5]	73.9 [73.2, 74.7]	71.8 [70.4, 73.1]	69.8 [68.4, 71.2]	70.6 [68.4, 72.8]	64.9 [62.5, 67.3]	77.4 [75.0, 79.6]	68.6 [65.8, 71.3]	74.4 [71.4, 77.3]	70.8 [69.8, 71.8]
Age (index screen)	50‐59	71.6 [71.3, 71.8]	71.6 [71.3, 71.8]	73.0 [72.1, 73.9]	72.1 [70.6, 73.5]	70.2 [68.2, 72.1]	71.1 [68.5, 73.5]	63.0 [60.1, 65.8]	76.9 [74.1, 79.5]	67.4 [64.0, 70.6]	75.2 [71.8, 78.5]	70.0 [68.9, 71.2]
	60‐69	78.0 [77.7, 78.2]	78.5 [78.2, 78.7]	76.6 [75.2, 78.0]	70.3 [66.9, 73.6]	69.4 [67.3, 71.5]	69.3 [64.7, 73.7]	70.4 [65.7, 74.8]	78.8 [74.1, 83.0]	71.5 [66.4, 76.2]	71.7 [64.6, 78.0]	73.3 [71.3, 75.2]
Urban/Rural residence	Urban	74.8 [74.6, 75.0]	75.2 [75.1, 75.4]	73.9 [73.2, 74.7]	71.9 [70.5, 73.2]	70.0 [68.6, 71.4]	70.5 [68.2, 72.6]	64.8 [62.3, 67.2]	78.2 [75.6, 80.6]	70.9 [67.9, 73.9]	74.4 [71.3, 77.3]	71.1 [70.0, 72.1]
	Rural	69.1 [68.7, 69.6]	69.2 [68.7, 69.7]	84.2 [60.4, 96.6]	65.9 [54.8, 75.8]	57.4 [43.2, 70.8]	100.0 [66.4, 100.0]	69.7 [51.3, 84.4]	72.8 [65.9, 79.0]	59.7 [53.2, 66.1]	100.0 [2.5, 100.0]	66.6 [61.9, 71.1]
Income quintile	1 (lowest)	70.8 [70.4, 71.3]	70.8 [70.3, 71.3]	72.8 [71.3, 74.3]	71.0 [68.6, 73.4]	69.1 [66.4, 71.8]	71.5 [65.1, 77.3]	58.8 [52.7, 64.6]	74.3 [66.2, 81.3]	66.4 [58.3, 73.9]	71.9 [66.2, 77.1]	70.1 [68.0, 72.2]
	2	73.4 [73.0, 73.7]	73.7 [73.3, 74.1]	73.7 [72.1, 75.3]	71.5 [68.8, 74.0]	70.2 [67.7, 72.6]	73.2 [68.2, 77.8]	68.4 [62.7, 73.8]	82.7 [76.2, 88.0]	63.2 [55.5, 70.4]	74.3 [68.8, 79.3]	69.0 [66.8, 71.1]
	3	74.3 [73.9, 74.6]	74.5 [74.1, 74.9]	75.3 [73.6, 76.9]	72.7 [69.6, 75.6]	70.8 [67.6, 73.9]	68.0 [61.8, 73.8]	66.0 [60.5, 71.2]	74.8 [69.0, 80.0]	69.9 [63.1, 76.0]	80.2 [73.3, 86.1]	70.7 [68.4, 73.0]
	4	74.9 [74.6, 75.2]	75.1 [74.8, 75.5]	74.8 [72.8, 76.7]	70.9 [66.9, 74.7]	69.7 [65.1, 73.9]	69.9 [65.0, 74.6]	67.9 [62.5, 73.0]	77.6 [72.5, 82.2]	71.1 [64.9, 76.7]	76.8 [66.2, 85.4]	72.6 [70.2, 74.9]
	5 (highest)	75.9 [75.5, 76.2]	76.2 [75.9, 76.5]	73.3 [71.2, 75.4]	74.5 [69.4, 79.1]	68.3 [62.4, 73.8]	70.6 [66.4, 74.6]	63.3 [57.9, 68.5]	78.3 [74.1, 82.0]	69.4 [64.3, 74.2]	63.6 [47.8, 77.6]	72.7 [70.3, 75.0]
# Major ADG's	0	79.9 [79.7, 80.1]	80.2 [80.0, 80.4]	79.8 [78.9, 80.7]	76.8 [75.1, 78.4]	75.4 [73.5, 77.2]	77.4 [74.6, 80.1]	71.1 [68.0, 74.2]	83.4 [80.6, 86.0]	77.7 [74.2, 81.0]	79.9 [76.0, 83.4]	76.8 [75.5, 78.0]
	1	80.5 [80.2, 80.8]	80.7 [80.4, 81.0]	82.3 [80.9, 83.7]	80.3 [77.7, 82.7]	75.8 [73.2, 78.3]	77.6 [73.4, 81.5]	75.5 [70.9, 79.8]	83.1 [78.7, 87.0]	71.0 [65.4, 76.2]	83.1 [77.3, 87.9]	77.5 [75.6, 79.3]
	2	80.3 [79.8, 80.8]	80.5 [80.0, 81.0]	81.4 [78.8, 83.9]	76.9 [71.8, 81.5]	76.2 [71.5, 80.5]	71.4 [63.6, 78.4]	75.8 [65.7, 84.2]	75.8 [65.9, 84.0]	69.8 [59.6, 78.7]	86.6 [76.0, 93.7]	80.7 [77.5, 83.5]
	3+	79.7 [78.8, 80.5]	79.7 [78.8, 80.5]	81.4 [76.7, 85.5]	80.0 [71.9, 86.6]	80.9 [73.8, 86.8]	73.1 [59.0, 84.4]	72.2 [54.8, 85.8]	82.4 [65.5, 93.2]	82.4 [65.5, 93.2]	80.8 [60.6, 93.4]	78.1 [72.0, 83.4]
	Unknown	8.4 [8.0, 8.7]	8.3 [8.0, 8.7]	8.9 [7.3, 10.6]	5.5 [3.4, 8.4]	6.0 [3.8, 9.1]	5.4 [2.4, 10.4]	6.6 [3.4, 11.2]	17.6 [10.9, 26.1]	17.6 [11.5, 25.2]	3.9 [0.8, 11.1]	9.2 [7.3, 11.5]
Index screen result	Normal	74.6 [74.4, 74.7]	74.9 [74.7, 75.1]	74.3 [73.5, 75.1]	72.2 [70.8, 73.6]	70.3 [68.8, 71.8]	71.5 [69.2, 73.7]	64.9 [62.4, 67.3]	78.2 [75.8, 80.5]	68.8 [65.9, 71.6]	75.3 [72.2, 78.3]	71.4 [70.3, 72.4]
	Abnormal	66.6 [66.0, 67.3]	66.8 [66.1, 67.5]	67.0 [63.3, 70.6]	67.0 [62.0, 71.8]	65.4 [60.4, 70.1]	59.4 [50.3, 68.0]	64.7 [52.2, 75.9]	66.3 [55.5, 76.0]	66.7 [55.9, 76.3]	60.0 [45.2, 73.6]	64.8 [60.9, 68.5]
Prior screening	None	44.4 [43.6, 45.1]	43.3 [42.5, 44.2]	46.4 [42.9, 49.9]	52.6 [48.1, 57.1]	48.9 [45.6, 52.1]	52.0 [44.8, 59.2]	40.3 [33.0, 48.0]	61.2 [51.1, 70.6]	42.9 [33.5, 52.6]	44.4 [33.4, 55.9]	46.1 [42.8, 49.5]
	Yes	76.0 [75.8, 76.2]	76.2 [76.0, 76.4]	75.8 [75.0, 76.6]	74.2 [72.8, 75.6]	76.0 [74.4, 77.5]	73.0 [70.7, 75.2]	68.0 [65.5, 70.5]	78.8 [76.4, 81.0]	71.4 [68.6, 74.2]	77.6 [74.5, 80.5]	73.8 [72.8, 74.8]
# PCP visits	0	64.4 [62.9, 65.9]	65.5 [64.0, 67.1]	50.8 [43.2, 58.4]	54.9 [40.3, 68.9]	71.4 [41.9, 91.6]	75.0 [34.9, 96.8]	44.4 [25.5, 64.7]	58.3 [36.6, 77.9]	58.8 [32.9, 81.6]	100.0 [29.2, 100.0]	59.2 [49.8, 68.0]
	1‐4	70.2 [69.8, 70.6]	70.9 [70.5, 71.3]	63.2 [60.9, 65.3]	64.4 [60.3, 68.3]	64.1 [56.6, 71.1]	58.7 [50.0, 67.0]	56.3 [50.2, 62.3]	73.9 [68.2, 79.2]	65.5 [59.5, 71.2]	64.9 [53.2, 75.5]	65.7 [63.0, 68.3]
	5‐9	74.7 [74.4, 75.0]	75.3 [75.0, 75.6]	71.9 [70.4, 73.3]	70.5 [67.9, 73.0]	65.0 [61.4, 68.5]	68.8 [63.7, 73.5]	65.1 [60.7, 69.2]	78.0 [73.8, 81.7]	70.1 [65.3, 74.6]	70.8 [63.8, 77.0]	69.6 [67.7, 71.5]
	10‐14	76.0 [75.7, 76.3]	76.2 [75.9, 76.6]	76.7 [75.1, 78.1]	75.1 [72.5, 77.5]	71.9 [68.9, 74.7]	72.8 [68.3, 77.0]	67.2 [62.2, 71.9]	81.3 [76.5, 85.6]	70.3 [63.9, 76.1]	72.5 [66.0, 78.4]	73.7 [71.6, 75.8]
	15+	74.6 [74.3, 74.9]	74.5 [74.1, 74.8]	80.1 [78.8, 81.4]	73.9 [71.4, 76.3]	71.0 [69.1, 72.9]	72.4 [69.1, 75.5]	70.1 [65.1, 74.7]	77.1 [71.8, 81.9]	68.8 [62.5, 74.6]	79.3 [74.8, 83.4]	73.1 [71.3, 74.8]
Years of residence in Canada^a^	<5	64.7 [62.8, 66.6]	NA	59.9 [55.2, 64.4]	67.8 [62.8, 72.6]	62.7 [58.6, 66.7]	67.8 [60.4, 74.5]	50.7 [38.9, 62.4]	72.7 [63.9, 80.4]	72.3 [65.5, 78.5]	67.7 [48.6, 83.3]	65.2 [60.5, 69.7]
	5‐9	67.2 [65.9, 68.5]		65.0 [62.3, 67.6]	70.5 [66.4, 74.4]	68.6 [65.7, 71.4]	70.6 [65.6, 75.2]	57.6 [51.4, 63.5]	81.3 [74.7, 86.7]	63.2 [55.9, 70.2]	64.7 [50.1, 77.6]	66.7 [63.7, 69.6]
	10‐19	73.3 [72.7, 73.9]		75.0 [74.1, 75.9]	72.5 [70.7, 74.3]	71.6 [69.6, 73.5]	70.5 [67.4, 73.4]	66.2 [63.1, 69.1]	77.9 [74.1, 81.4]	71.0 [66.3, 75.4]	75.9 [71.2, 80.2]	72.7 [71.3, 74.1]
	20+	73.5 [72.5, 74.4]		77.8 [76.0, 79.5]	71.9 [69.1, 74.7]	74.2 [69.6, 78.4]	73.1 [67.4, 78.3]	72.8 [66.6, 78.5]	76.6 [72.7, 80.3]	66.8 [61.7, 71.6]	74.9 [70.3, 79.1]	70.6 [68.8, 72.3]

ADG, aggregate diagnosis group; CMHT, China, Macau, Hong Kong, Taiwan; PCP, primary care physician.

a Years of residence in Canada for the “Population” column refers to the pooled group of all immigrants.

### 3.3 Characteristics and screening participation of recent immigrants

Table 7 provides the characteristics and participation rates by birth country for immigrant women with <10 years of residence in Canada. This analysis was limited to the 8 most common birth countries among recent immigrants, which represented more than 80% of all recent immigrants. Screening participation rates were not calculated for cells with <10 women, identified in the table as “NC” (not calculated). The most common birth countries represented within this population are nearly identical to the most common countries identified among all immigrant women (Table 3) with the only difference being a substitution of the Former USSR in the recent immigrant group for Vietnam in the total immigrant population.

**Table 7:** Characteristics of and participation rates for recent immigrants (<10 years duration in Canada) for most common countries of birth among recent immigrants

Variable	Subgroup	CMHT (N = 5021)		India (N = 4072)		Philippines (N = 2329)		South Korea (N = 1006)		Iran (N = 930)	
		N (%)	PR (%)	N (%)	PR (%)	N (%)	PR (%)	N (%)	PR (%)	N (%)	PR (%)
Age	50‐59	3956 (78.8%)	36.1 [34.6, 37.7]	2139 (52.5%)	41.9 [39.8, 44.0]	1926 (82.7%)	41.7 [39.5, 44.0]	923 (91.7%)	28.8 [25.9, 31.9]	634 (68.2%)	55.4 [51.4, 59.3]
	60‐69	1065 (21.2%)	31.4 [28.6, 34.2]	1933 (47.5%)	32.0 [29.9, 34.1]	403 (17.3%)	36.0 [31.3, 40.9]	83 (8.3%)	27.7 [18.4, 38.6]	296 (31.8%)	54.1 [48.2, 59.8]
Urban/rural residence	Urban	4998 (99.5%)	35.2 [33.9, 36.5]	4012 (98.5%)	37.3 [35.8, 38.8]	2283 (98.0%)	41.0 [38.9, 43.0]	997 (99.1%)	28.3 [25.5, 31.2]	930 (100.0%)	54.9 [51.7, 58.2]
	Rural	22 (0.4%)	22.7 [7.8, 45.4]	60 (1.5%)	30.0 [18.8, 43.2]	46 (2.0%)	30.4 [17.7, 45.8]	9 (0.9%)	NC	0 (0.0%)	NC
	Unknown	<5 (<0.1%)	NC	0 (0.0%)	NC	0 (0.0%)	NC	0 (0.0%)	NC	0 (0.0%)	NC
Income quintile	1 (lowest)	1570 (31.3%)	34.4 [32.0, 36.8]	1098 (27.0%)	37.1 [34.2, 40.0]	803 (34.5%)	40.0 [36.6, 43.5]	197 (19.6%)	24.4 [18.5, 31.0]	136 (14.6%)	50.0 [41.3, 58.7]
	2	1143 (22.8%)	37.4 [34.6, 40.3]	1489 (36.6%)	38.1 [35.6, 40.6]	697 (29.9%)	39.2 [35.5, 42.9]	178 (17.7%)	29.8 [23.2, 37.1]	186 (20.0%)	57.0 [49.5, 64.2]
	3	887 (17.7%)	35.2 [32.0, 38.4]	858 (21.1%)	37.3 [34.1, 40.6]	427 (18.3%)	43.8 [39.0, 48.6]	202 (20.1%)	35.1 [28.6, 42.2]	108 (11.6%)	58.3 [48.5, 67.7]
	4	646 (12.9%)	33.0 [29.4, 36.7]	386 (9.5%)	33.7 [29.0, 38.6]	242 (10.4%)	44.2 [37.9, 50.7]	220 (21.9%)	32.3 [26.1, 38.9]	210 (22.6%)	55.7 [48.7, 62.5]
	5 (highest)	702 (14.0%)	35.6 [32.1, 39.3]	239 (5.9%)	37.2 [31.1, 43.7]	148 (6.4%)	39.2 [31.3, 47.5]	181 (18.0%)	24.9 [18.7, 31.8]	241 (25.9%)	56.0 [49.5, 62.4]
	Unknown	73 (1.5%)	28.8 [18.8, 40.6]	<5 (<0.1%)	NC	12 (0.5%)	25.0 [5.5, 57.2]	28 (2.8%)	3.6 [0.1, 18.3]	49 (5.3%)	44.9 [30.7, 59.8]
Education level	None	48 (1.0%)	22.9 [12.0, 37.3]	2015 (49.5%)	31.2 [29.1, 33.2]	37 (1.6%)	32.4 [18.0, 49.8]	8 (0.8%)	50.0 [15.7, 84.3]	18 (1.9%)	55.6 [30.8, 78.5]
	Secondary or less	2477 (49.3%)	35.7 [33.8, 37.6]	1417 (34.8%)	41.8 [39.3, 44.5]	246 (10.6%)	31.3 [25.6, 37.5]	316 (31.4%)	26.9 [22.1, 32.1]	394 (42.4%)	55.8 [50.8, 60.8]
	Diploma/Certificate/Some University	1525 (30.4%)	36.3 [33.9, 38.8]	127 (3.1%)	47.2 [38.3, 56.3]	440 (18.9%)	40.7 [36.1, 45.4]	198 (19.7%)	28.8 [22.6, 35.6]	124 (13.3%)	62.1 [52.9, 70.7]
	Undergraduate	750 (14.9%)	33.3 [30.0, 36.8]	306 (7.5%)	48.4 [42.6, 54.1]	1453 (62.4%)	42.3 [39.7, 44.8]	368 (36.6%)	29.3 [24.7, 34.3]	262 (28.2%)	55.0 [48.7, 61.1]
	Graduate	221 (4.4%)	29.4 [23.5, 35.9]	207 (5.1%)	41.1 [34.3, 48.1]	153 (6.6%)	43.8 [35.8, 52.0]	116 (11.5%)	30.2 [22.0, 39.4]	132 (14.2%)	45.5 [36.8, 54.3]
Canadian language skill	English/French	1080 (21.5%)	35.9 [33.1, 38.9]	975 (23.9%)	42.7 [39.5, 45.8]	2171 (93.2%)	40.9 [38.8, 43.0]	513 (51.0%)	28.5 [24.6, 32.6]	616 (66.2%)	54.2 [50.2, 58.2]
	None	3941 (78.5%)	34.9 [33.4, 36.4]	3097 (76.1%)	35.5 [33.8, 37.2]	158 (6.8%)	39.2 [31.6, 47.3]	493 (49.0%)	29.0 [25.0, 33.2]	314 (33.8%)	56.4 [50.7, 61.9]
Applicant type	Principal	2017 (40.2%)	36.7 [34.6, 38.9]	925 (22.7%)	35.0 [32.0, 38.2]	1725 (74.1%)	39.7 [37.3, 42.0]	292 (29.0%)	30.5 [25.3, 36.1]	336 (36.1%)	52.4 [46.9, 57.8]
	Dependent	3004 (59.8%)	34.1 [32.4, 35.8]	3147 (77.3%)	37.8 [36.1, 39.5]	604 (25.9%)	43.9 [39.9, 47.9]	714 (71.0%)	28.0 [24.7, 31.5]	594 (63.9%)	56.4 [52.3, 60.4]
Immigration class	Economic	3126 (62.3%)	33.8 [32.1, 35.5]	372 (9.1%)	42.5 [37.4, 47.7]	1775 (76.2%)	41.9 [39.6, 44.3]	847 (84.2%)	29.0 [26.0, 32.2]	537 (57.7%)	54.2 [49.9, 58.5]
	Family	1682 (33.5%)	37.0 [34.7, 39.4]	3647 (89.6%)	36.6 [35.0, 38.1]	519 (22.3%)	36.8 [32.6, 41.1]	133 (13.2%)	27.1 [19.7, 35.5]	272 (29.2%)	56.3 [50.1, 62.2]
	Refugee	138 (2.7%)	41.3 [33.0, 50.0]	35 (0.9%)	48.6 [31.4, 66.0]	7 (0.3%)	NC	11 (1.1%)	36.4 [10.9, 69.2]	92 (9.9%)	57.6 [46.9, 67.9]
	Other	75 (1.5%)	37.3 [26.4, 49.3]	18 (0.4%)	33.3 [13.3, 59.0]	28 (1.2%)	50.0 [30.6, 69.4]	15 (1.5%)	20.0 [4.3, 48.1]	29 (3.1%)	48.3 [29.4, 67.5]
# Major ADG's	0	3693 (73.6%)	32.1 [30.6, 33.6]	2427 (59.6%)	34.8 [32.9, 36.7]	1624 (69.7%)	38.5 [36.2, 41.0]	759 (75.4%)	24.9 [21.9, 28.1]	570 (61.3%)	51.1 [46.9, 55.2]
	1	892 (17.8%)	44.6 [41.3, 48.0]	1088 (26.7%)	39.7 [36.8, 42.7]	478 (20.5%)	46.9 [42.3, 51.4]	160 (15.9%)	38.1 [30.6, 46.1]	221 (23.8%)	59.3 [52.5, 65.8]
	2	229 (4.6%)	44.1 [37.6, 50.8]	314 (7.7%)	42.7 [37.1, 48.4]	126 (5.4%)	46.0 [37.1, 55.1]	47 (4.7%)	48.9 [34.1, 63.9]	88 (9.5%)	58.0 [47.0, 68.4]
	3+	56 (1.1%)	37.5 [24.9, 51.5]	120 (2.9%)	47.5 [38.3, 56.8]	35 (1.5%)	42.9 [26.3, 60.6]	8 (0.8%)	NC	20 (2.2%)	75.0 [50.9, 91.3]
	Unknown	151 (3.0%)	39.1 [31.2, 47.3]	123 (3.0%)	37.4 [28.8, 46.6]	66 (2.8%)	39.4 [27.6, 52.2]	32 (3.2%)	34.4 [18.6, 53.2]	31 (3.3%)	74.2 [55.4, 88.1]
# PCP visits	Median [IQR]	6.0 [2.0‐11.0]		13.0 [7.0‐19.0]		8.0 [5.0‐13.0]		5.0 [1.0‐9.0]		11.0 [6.0‐17.0]	
	0	769 (15.3%)	6.1 [4.5, 8.0]	129 (3.2%)	10.9 [6.1, 17.5]	120 (5.2%)	10.8 [5.9, 17.8]	211 (21.0%)	5.2 [2.6, 9.1]	57 (6.1%)	8.8 [2.9, 19.3]
	1‐4	1310 (26.1%)	27.4 [25.0, 29.9]	425 (10.4%)	22.8 [18.9, 27.1]	442 (19.0%)	29.4 [25.2, 33.9]	271 (26.9%)	26.2 [21.1, 31.9]	119 (12.8%)	37.0 [28.3, 46.3]
	5‐9	1425 (28.4%)	42.5 [39.9, 45.1]	847 (20.8%)	33.4 [30.2, 36.7]	772 (33.1%)	43.3 [39.7, 46.8]	279 (27.7%)	33.7 [28.2, 39.6]	219 (23.5%)	57.1 [50.2, 63.7]
	10+	1517 (30.2%)	49.6 [47.0, 52.1]	2671 (65.6%)	41.9 [40.1, 43.8]	995 (42.7%)	47.4 [44.3, 50.6]	245 (24.4%)	46.1 [39.8, 52.6]	535 (57.5%)	63.0 [58.7, 67.1]

Variable	Subgroup	United States (N = 849)		United Kingdom (N = 599)		Former USSR State (N = 473)		Other Immigrants (N = 3216)	
		N (%)	PR (%)	N (%)	PR (%)	N (%)	PR (%)	N (%)	PR (%)
Age	50‐59	543 (64.0%)	41.3 [37.1, 45.5]	465 (77.6%)	52.5 [47.8, 57.1]	347 (73.4%)	38.0 [32.9, 43.4]	2362 (73.4%)	40.4 [38.4, 42.4]
	60‐69	306 (36.0%)	43.8 [38.2, 49.6]	134 (22.4%)	53.0 [44.2, 61.7]	126 (26.6%)	27.0 [19.5, 35.6]	854 (26.6%)	36.2 [33.0, 39.5]
Urban/Rural residence	Urban	636 (74.9%)	44.2 [40.3, 48.1]	496 (82.8%)	52.8 [48.3, 57.3]	451 (95.3%)	34.6 [30.2, 39.2]	3027 (94.1%)	39.3 [37.6, 41.1]
	Rural	213 (25.1%)	36.2 [29.7, 43.0]	103 (17.2%)	51.5 [41.4, 61.4]	22 (4.7%)	45.5 [24.4, 67.8]	188 (5.8%)	38.8 [31.8, 46.2]
	Unknown	0 (0.0%)	NC	0 (0.0%)	NC	0 (0.0%)	NC	<5 (<0.2%)	NC
Income quintile	1 (lowest)	144 (17.0%)	43.1 [34.8, 51.6]	61 (10.2%)	62.3 [49.0, 74.4]	136 (28.8%)	28.7 [21.3, 37.1]	943 (29.3%)	38.3 [35.2, 41.5]
	2	151 (17.8%)	41.7 [33.8, 50.0]	70 (11.7%)	37.1 [25.9, 49.5]	109 (23.0%)	39.4 [30.2, 49.3]	708 (22.0%)	38.8 [35.2, 42.5]
	3	153 (18.0%)	39.9 [32.1, 48.1]	104 (17.4%)	47.1 [37.2, 57.2]	92 (19.5%)	32.6 [23.2, 43.2]	587 (18.3%)	41.2 [37.2, 45.3]
	4	162 (19.1%)	39.5 [31.9, 47.5]	151 (25.2%)	51.7 [43.4, 59.9]	77 (16.3%)	42.9 [31.6, 54.6]	480 (14.9%)	37.5 [33.2, 42.0]
	5 (highest)	231 (27.2%)	44.6 [38.1, 51.2]	211 (35.2%)	58.3 [51.3, 65.0]	51 (10.8%)	35.3 [22.4, 49.9]	470 (14.6%)	43.0 [38.5, 47.6]
	Unknown	8 (0.9%)	NC	<5 (<0.8%)	NC	8 (1.7%)	NC	28 (0.9%)	14.3 [4.0, 32.7]
Education level	None	10 (1.2%)	20.0 [2.5, 55.6]	67 (11.2%)	55.2 [42.6, 67.4]	<5 (<1.1%)	NC	231 (7.2%)	32.0 [26.1, 38.5]
	Secondary or less	169 (19.9%)	35.5 [28.3, 43.2]	156 (26.0%)	48.7 [40.6, 56.8]	37 (7.8%)	21.6 [9.8, 38.2]	1161 (36.1%)	36.6 [33.8, 39.5]
	Diploma/Certificate/Some University	160 (18.8%)	36.3 [28.8, 44.2]	239 (39.9%)	53.1 [46.6, 59.6]	121 (25.6%)	27.3 [19.6, 36.1]	799 (24.8%)	39.8 [36.4, 43.3]
	Undergraduate	260 (30.6%)	43.8 [37.7, 50.1]	94 (15.7%)	51.1 [40.5, 61.5]	217 (45.9%)	41.0 [34.4, 47.9]	688 (21.4%)	43.0 [39.3, 46.8]
	Graduate	250 (29.4%)	49.6 [43.2, 56.0]	43 (7.2%)	62.8 [46.7, 77.0]	97 (20.5%)	36.1 [26.6, 46.5]	337 (10.5%)	44.8 [39.4, 50.3]
Canadian language skill	English/French	845 (99.5%)	42.1 [38.8, 45.5]	597 (99.7%)	52.6 [48.5, 56.7]	252 (53.3%)	38.5 [32.5, 44.8]	2408 (74.9%)	40.6 [38.6, 42.6]
	None	<5 (<0.6%)	NC	<5 (<0.8%)	NC	221 (46.7%)	31.2 [25.2, 37.8]	808 (25.1%)	35.4 [32.1, 38.8]
Applicant type	Principal	608 (71.6%)	43.1 [39.1, 47.1]	291 (48.6%)	47.8 [41.9, 53.7]	268 (56.7%)	40.3 [34.4, 46.4]	1867 (58.1%)	38.5 [36.3, 40.8]
	Dependent	241 (28.4%)	39.8 [33.6, 46.3]	308 (51.4%)	57.1 [51.4, 62.7]	205 (43.3%)	28.3 [22.2, 35.0]	1349 (41.9%)	40.4 [37.8, 43.1]
Immigration class	Economic	421 (49.6%)	44.7 [39.8, 49.5]	422 (70.5%)	52.8 [48.0, 57.7]	252 (53.3%)	33.7 [27.9, 39.9]	1243 (38.7%)	40.3 [37.6, 43.1]
	Family	381 (44.9%)	40.9 [36.0, 46.1]	153 (25.5%)	52.3 [44.1, 60.4]	176 (37.2%)	40.3 [33.0, 48.0]	1263 (39.3%)	38.1 [35.4, 40.8]
	Refugee	0 (0.0%)	NC	0 (0.0%)	NC	22 (4.7%)	18.2 [5.2, 40.3]	561 (17.4%)	40.1 [36.0, 44.3]
	Other	47 (5.5%)	29.8 [17.3, 44.9]	24 (4.0%)	50.0 [29.1, 70.9]	23 (4.9%)	26.1 [10.2, 48.4]	149 (4.6%)	38.3 [30.4, 46.6]
# Major ADG's	0	500 (58.9%)	39.8 [35.5, 44.2]	387 (64.6%)	52.7 [47.6, 57.8]	333 (70.4%)	33.0 [28.0, 38.4]	2090 (65.0%)	36.4 [34.3, 38.5]
	1	228 (26.9%)	46.9 [40.3, 53.6]	151 (25.2%)	57.0 [48.7, 65.0]	91 (19.2%)	44.0 [33.6, 54.8]	722 (22.5%)	44.3 [40.7, 48.0]
	2	65 (7.7%)	52.3 [39.5, 64.9]	28 (4.7%)	42.9 [24.5, 62.8]	29 (6.1%)	27.6 [12.7, 47.2]	226 (7.0%)	50.0 [43.3, 56.7]
	3+	20 (2.4%)	25.0 [8.7, 49.1]	13 (2.2%)	61.5 [31.6, 86.1]	11 (2.3%)	45.5 [16.7, 76.6]	84 (2.6%)	36.9 [26.6, 48.1]
	Unknown	36 (4.2%)	36.1 [20.8, 53.8]	20 (3.3%)	25.0 [8.7, 49.1]	9 (1.9%)	NC	94 (2.9%)	41.5 [31.4, 52.1]
# PCP visits	Median [IQR]	7.0 [4.0‐12.0]		7.0 [3.0‐11.0]		7.0 [3.0‐12.0]		8.0 [3.0‐14.0]	
	0	51 (6.0%)	15.7 [7.0, 28.6]	43 (7.2%)	23.3 [11.8, 38.6]	45 (9.5%)	15.6 [6.5, 29.5]	292 (9.1%)	14.4 [10.6, 18.9]
	1‐4	216 (25.4%)	31.9 [25.8, 38.6]	156 (26.0%)	41.0 [33.2, 49.2]	127 (26.8%)	28.3 [20.7, 37.0]	688 (21.4%)	31.4 [27.9, 35.0]
	5‐9	263 (31.0%)	46.8 [40.6, 53.0]	203 (33.9%)	58.6 [51.5, 65.5]	133 (28.1%)	35.3 [27.3, 44.1]	846 (26.3%)	40.8 [37.4, 44.2]
	10+	319 (37.6%)	49.5 [43.9, 55.2]	197 (32.9%)	61.9 [54.8, 68.7]	168 (35.5%)	45.2 [37.6, 53.1]	1390 (43.2%)	47.6 [44.9, 50.2]

ADG, aggregate diagnosis group; CMHT, China, Macau, Hong Kong, Taiwan; IQR, inter‐quartile range; N, sample size; NC, not calculated due to sample size <10; PCP, primary care physician; PR, participation rate; USSR, Union of Soviet Socialist Republics.

Recent immigrants were more commonly aged 50‐59, with the notable exception recent Indian immigrants who were almost evenly split among age groups. Screening participation rates were lower in the older age group for several immigrant groups (women from CMHT, India, Philippines, Former USSR and Other Immigrants); however, in other groups, there appeared to be little relationship. There was a considerable range in Canadian language fluency with women from CMHT and India having >75% with no competence in English or French, while others reported near 100% fluency. Language competence did not seem to associate with screening participation in several of the immigrant groups (CMHT, the Philippines, South Korea, Iran) while women from the India, Former USSR, and Other Immigrant groups showed lower screening participation among women with no Canadian language competency. Education level at the time of landing similarly showed strong variation across study groups. Chinese immigrants reported 50.3% having secondary school education or less; nearly half of recent Indian immigrants reported no formal education and an additional 34.8% reported secondary school or less. For immigrant women from the Philippines, US, and the Former USSR, the percentage of women with undergraduate or graduate degrees was above 60% suggesting highly educated groups. Curiously, 11.2% of recent immigrants from the UK reported no formal education which may represent a data quality issue; the majority of these women were identified as dependent immigrants within the immigration data. Indian immigrants and Other Immigrants also showed a comparatively higher percentage of women with no prior education. There was a strong association in these 2 groups between reporting no prior education and older age, no Canadian language proficiency, and immigrating as a family class or refugee immigrant (data not shown). The relationship between education level and screening participation was not uniform across immigrant groups.

Screening participation rates were higher among refugee immigrants compared to either economic or family class immigrants for immigrants from CMHT, India, and South Korea. In all 3 of these groups, however, the number of women immigrating under this class represented a very small proportion of the immigrant population. Among immigrants from the Former USSR, refugee class immigrants showed the lowest participation rates; however, this group was comprised of only 22 women, and thus, the participation rate is highly imprecise.

The median number of PCP visits was lower for women from South Korea (5.0) and CMHT (6.0) and much higher for women from India (13.0) and Iran (11.0). The percentage of South Korean (21.0%) and CMHT (15.3%) recent immigrants that had no PCP visits in the 2‐year look‐back period was higher than in the other groups. As with the analysis on the entire cohort, the recent immigrant analysis revealed a strong positive relationship between screening participation and number of PCP visits. In all immigrant groups, the women with no recent PCP visits had the lowest screening rates.

Our analysis to identify independent predictors of breast screening participation among the most recent immigrants identified only the number of PCP visits as a significant predictor within all immigrant groups. Compared to women who had 10 or more PCP visits, those with no recent PCP visits showed adjusted relative risks (ARRs) in the range from 0.11 to 0.37 (Table 8) indicating much lower screening. ARRs increased in each immigrant group with the number of PCP visits. Older age (60‐69 vs 50‐59) was associated with less screening participation in women from CMHT, India, the Philippines, the Former USSR, and Other Immigrants. Among women from India, US, Former USSR, and Other Immigrants, those with lower education levels tended to screen less in comparison with women with graduate education. The Former USSR group had <10 women reporting no formal education, and thus, this group was pooled with the “secondary school or less” group for this analysis. Thus the ARR for this group needs to be interpreted differently than for the other immigrant populations. The Former USSR group was the only group for which the immigrant class variable was significantly associated with participation with family class immigrants demonstrating greater participation compared to economic migrants (ARR = 1.57). Although considered in the analysis, Canadian language proficiency at the time of landing, rural residence, and the number of major ADGs was not identified as being significantly associated with participation in any of the immigrant groups.

**Table 8:** Adjusted rate ratios for identified predictors of screening among recent immigrants (<10 years duration in Canada)

Variable	Subgroup	CMHT	India	Philippines	South Korea	Iran	US	UK	Former USSR	Other Immigrants
Age	60‐69 vs 50‐59	0.76 (0.69, 0.84)	0.79 (0.72, 0.85)	0.83 (0.73, 0.96)	—	—	—	—	0.54 (0.38, 0.75)	0.90 (0.81, 0.99)
Income quintile	Q1 (Lowest) vs Q5	—	—	—	—	—	—	1.09 (0.88, 1.35)	—	—
	Q2 vs Q5	—	—	—	—	—	—	0.64 (0.47, 0.88)	—	—
	Q3 vs Q5	—	—	—	—	—	—	0.82 (0.66, 1.03)	—	—
	Q4 vs Q5	—	—	—	—	—	—	0.87 (0.72, 1.06)	—	—
# PCP visits	None vs 10+	0.12 (0.09, 0.16)	0.25 (0.15, 0.41)	0.23 (0.14, 0.38)	0.11 (0.06, 0.20)	0.14 (0.06, 0.32)	0.32 (0.17, 0.60)	0.37 (0.22, 0.65)	0.33 (0.16, 0.66)	0.29 (0.22, 0.39)
	1‐4 vs 10+	0.54 (0.48, 0.59)	0.52 (0.43, 0.62)	0.61 (0.52, 0.72)	0.57 (0.45, 0.72)	0.59 (0.46, 0.75)	0.64 (0.51, 0.80)	0.65 (0.52, 0.80)	0.58 (0.42, 0.79)	0.63 (0.56, 0.71)
	5‐9 vs 10+	0.83 (0.77, 0.90)	0.78 (0.70, 0.86)	0.91 (0.82, 1.01)	0.73 (0.59, 0.90)	0.91 (0.79, 1.03)	0.92 (0.78, 1.09)	0.93 (0.79, 1.08)	0.76 (0.57, 1.00)	0.83 (0.75, 0.91)
Immigrant class	Family vs Economic	—	—	—	—	—	—	—	1.57 (1.20, 2.05)	—
	Refugee vs Economic	—	—	—	—	—	—	—	0.73 (0.30, 1.77)	—
	Other vs Economic	—	—	—	—	—	—	—	0.99 (0.51, 1.91)	—
Education level	None vs Graduate	—	0.76 (0.64, 0.90)	—	—	—	0.41 (0.12, 1.46)	—	—	0.66 (0.53, 0.83)
	Secondary or less vs Graduate	—	1.00 (0.84, 1.19)	—	—	—	0.72 (0.57, 0.91)	—	0.67^a^ (0.36, 1.25)	0.77 (0.67, 0.88)
	Diploma/Certificate/Some University vs Graduate	—	1.22 (0.96, 1.55)	—	—	—	0.73 (0.58, 0.92)	—	0.65 (0.44, 0.98)	0.87 (0.76, 1.01)
	Undergraduate vs Graduate	—	1.2 (0.99, 1.46)	—	—	—	0.88 (0.73, 1.05)	—	1.12 (0.83, 1.51)	0.93 (0.81, 1.07)

“—” indicates no estimate available for this variable as this term was not retained in final model; CMHT, China, Macau, Hong Kong, Taiwan; PCP, primary care physician; Q, quintile; UK, United Kingdom; US, United States of America; vs, versus.

Other variables considered that were not predictive for any immigrant population were # of major aggregate diagnosis groups, rural residence and Canadian language ability at time of immigration. Some variables could not be considered in all groups due to sample sizes including rural residence within Korean immigrants and Canadian language ability among immigrants from USA and UK.

a Within Former USSR education categories “secondary or less” and “none” were grouped together due to small sample size.

## 4 DISCUSSION

Our study demonstrates that screening mammography participation rates in BC are lower for some immigrant subpopulations compared to nonimmigrant women. We identified variation in participation rates when women are grouped by both world region of birth and by individual countries of birth. Participation rates also varied within immigrant subpopulations according to age group, duration of residence in Canada, as well as other socio‐demographic variables. At the same time, the relationship between participation and these variables was not consistent across the immigrant populations.

To our knowledge, this is the first large population‐based study in Canada that has examined breast screening retention rates as an endpoint in comparing immigrant and nonimmigrant groups. In comparison with the participation rate analysis, we found less variation in screening retention rates across both world region and birth country groups. When the analysis was restricted to women with at least one mammogram within the program prior to the index screen, the variability in retention rates was reduced further (Table S3). Our retention rate analysis revealed less disparity with the nonimmigrant population for women from the Central Asian/Eastern European region compared to what was observed in the participation analysis. Indian immigrant women showed lower participation rates compared to nonimmigrant women; however, in the retention rate analysis that was restricted to those women with at least one screen prior to the index screen, the retention rates for the 2 groups were essentially identical. These findings of lower variation in retention rates across many of the groups we examined and less disparity with the nonimmigrant rate are possibly encouraging in that they may suggest that different groups of women, once attracted to programmatic screening, can be similarly retained.

Our participation results are consistent with prior Canadian studies that have reported lower breast cancer screening rates among immigrant women and specific immigrant subpopulations.^9^, ^10^, ^11^, ^24^ Recent data from Ontario, Canada^9^ demonstrated that among immigrant women, South Asians had the lowest breast screening utilization rate with the age 60‐69 group demonstrating lower screening than those aged 50‐59 years. Similar results were found for Indian immigrant women in our cohort. Consistent with our findings, Eastern European/Central Asian immigrant women in Ontario were also found to have among the lowest participation rates. Our results provide additional detail, showing that participation rates are consistently poor among all countries in this regional group. Prior Canadian studies have also noted that breast screening participation is strongly associated with duration of residence in Canada.^9^, ^10^, ^12^, ^24^ Although we observed this same association within many of the immigrant groups we examined, immigrants from Iran and the UK did not show a clear association. Further, when screening retention was examined, the association with duration of residence in Canada was less consistent across groups examined. While it was generally true that retention rates were lower among the most recent immigrants, the retention rates did not increase across the categories reflecting increased duration of residence in Canada in several groups.

Our analysis at the individual country level identified some screening patterns not reported in recent studies which examined data at the world region level. Screening participation and retention among South Korean immigrant women in our study was very low relative to nonimmigrants and other immigrant populations; among recent immigrants, this group also demonstrated some of the lowest participation and retention rates. Our findings also suggested that a significant proportion of this group had no apparent PCP visits in the look‐back period (15.2% overall and 21.0% of recent immigrants) and low proficiency in Canadian languages. As noted in a recent review article,^25^ there have been limited Canadian data reporting on breast screening rates for Filipino women who are BC's second largest population of screening‐age immigrant women. Thus, our study contributes important data on this population including a description of both screening patterns and characteristics of the screening‐age Filipino immigrant population in BC. Although overall participation for this group was similar to the East Asia/Pacific region rate, Filipino women residing in Canada for <10 years had a participation rate 14% lower than that of nonimmigrants; among women aged 60‐69 the rate was 19% lower. The characteristics of recent Filipino immigrants suggested a highly educated group with strong Canadian language proficiency, living almost entirely in urban areas; further, almost 95% of recent Filipino immigrants reported some PCP visits in the look‐back period. Thus, the information identified within the present study may help to support interventions and promotions within both of these populations.

There have been numerous studies of breast cancer screening among immigrant populations undertaken in other countries.^26^, ^27^, ^28^, ^29^, ^30^, ^31^, ^32^ Comparing results between studies is challenging as the birth country composition of immigrant populations vary significantly across countries, barriers to screening for immigrants in their adopted country may be different, and the health and cancer screening systems may also differ in significant ways. Acknowledging the challenges with comparing studies, findings have generally reported that screening rates among immigrant populations are lower than those among nonimmigrants. Among these studies, several have further assessed and reported a similar positive association between duration of residence in the adopted country and screening participation.^26^, ^30^, ^31^ A recent population‐based study from Norway reported that participation rates rose much more quickly with years of residence in Norway for women who immigrated from high‐income countries compared to those that immigrated from middle‐ or low‐income countries. Income level of the source country does not completely explain the patterns observed in our study as for example, South Korea showed a gradual increase in participation with duration of residence in Canada, similar to Indian or CMHT immigrants. Among studies that have reported screening rates for immigrants by region or country of birth, there are similarities to our findings of lower rates of screening among women from Eastern European/Central Asian, East Asian, and South Asian countries.^26^, ^30^

Our results provide further evidence of the strong association between screening participation and primary care physician (PCP) contact. Participation rates within each of our study groups generally increased with physician contact; the lowest rates were observed in the groups of patients that reported no PCP visits. Further, in our analysis of predictors of screening participation among recent immigrants, the number of PCP visits was the only variable associated with having been screened in all immigrant groups examined. For recent South Korean immigrant women with no PCP visits (21% of all recent South Korean immigrants), the participation rate was only 5.2% while for women with 10 or more PCP visits it was 46.1%.The results were nearly identical for recent Chinese immigrant women and similar for other immigrant groups. Generally, retention rates were higher for groups with more PCP contact; it is worth noting that <2% of each study group in the retention rate analysis had no PCP visits and thus their retention rates are highly imprecise. Despite using a number of definitions for PCP contact, the association with breast screening has been reported in a number of Canadian studies.^9^, ^10^, ^11^, ^24^, ^33^, ^34^ Generally, they have found recent contact with a PCP was associated with increased screening participation^9^, ^24^, ^33^, ^34^ or that a greater number of PCP visits were associated with higher participation rates.^9^

In Ontario, there has been considerable work examining the specific patient enrollment model (PEM) that attaches patients to a PCP and how this correlates with breast screening.^8^, ^9^, ^10^, ^11^ PEM's provide various models of rostering patients to individual or teams of physicians with differences in the model of remuneration for care provided to patients. In a recent study of breast screening among immigrants to Ontario, only 10% of women in their population‐based cohort were not enrolled in some kind of PEM.^9^ In BC, primary care is typically remunerated under a fee‐for‐service (FFS) model, and patients and physicians are not formally rostered together. Thus, our findings cannot be directly compared to studies that have shown that screening rates are generally improved among immigrant women rostered to PCP's with a PEM compared to those who are not.^9^, ^10^ However, recent research has attempted to better characterize the PCP population in BC by examining the variation in PCP practice style using available administrative data^35^ and suggests there is variation in the level of responsibility that fee‐for‐service PCPs assume for patients they see. Future work could thus assess the characteristics and practice style of the PCPs that immigrant and nonimmigrant women see and how these factors associate with screening uptake among eligible women.

Our study strengths include the use of population‐based, administrative data sets which permit the estimation of population screening rates and reduce the potential for selection bias. Thus, in contrast to studies that utilize survey methods, the present study is not affected by survey response bias or recall bias associated with the timing of the most recent mammogram reported by respondents. The data sets included information from diverse sources permitting the examination of screening rates by a variety of socio‐demographic and health variables. The immigration data included the specific country of birth of each immigrant woman in our cohort permitting us to examine screening indicators by country of birth rather than aggregate world regions alone.

Reliance on administrative data does, however, impose some limitations on our findings. Although our study aimed to compare screening participation and retention rates in immigrant and nonimmigrant women, women who immigrated to Canada prior to 1985 are included in the nonimmigrant group. Although the magnitude of this misclassification is not known, because long‐term immigrants tend to exhibit similar screening rates to nonimmigrants, our reported rates among immigrants are almost certainly lower than they would be had we been able to identify immigrants that landed in Canada prior to 1985 and include them in the immigrant group. The linkage of the immigration database to the provincial health client file to identify immigrants within the BC health system database was not performed directly by the research team and thus we could not directly assess the quality of the linked cases. The group responsible for the linkage, however, has significant expertise in record linkage and maintains the linked database infrastructure for a provincial, health data linkage research platform. We were also unable to examine immigrant women's screening, surgical and breast cancer histories prior to when they were represented in provincial data sets. Thus, women who developed breast cancer or had mastectomy surgeries outside of BC could not be removed from the screening‐eligible cohort. The unexpectedly high percentage of immigrants from the UK reporting no prior formal education may suggest that education status was not accurately captured for all women within the immigration data. As the majority of these women were identified as dependents (in place of principal applicants) in the immigration data, it is possible that this information is less relevant for this type of applicant and was not captured as accurately. Unfortunately, we were unable to conduct verifications of the data due to the administrative nature of its collection and thus this remains a limitation of our data. It is possible that the imperfect measurement of this variable could impact the model selection and our ability to detect education effects or the effects of other variables considered in the models; however, we cannot fully quantify the potential impact of this.

We were also limited in our ability to count primary care visits definitively for each cohort member. Although primary care in BC is mainly delivered by fee‐for‐service physicians, a smaller fraction of physicians are paid under an alternate payment program for which no service use data are available. The implication is some patients who appeared to have no primary care encounters may in fact have seen a physician paid through alternative payments. Only 5.9% of the total cohort had no physician visits within our look‐back period and thus the affected group did not comprise a significant portion of the total cohort. We relied on an ecological variable for socioeconomic status (income quintile) based on the cohort member's postal code of residence which may not accurately reflect their true income or socioeconomic status. The area‐based income quintile available within our data sets was also derived using information collected from the 2006 Canadian census. It is possible that some neighborhood incomes have changed between 2006 and our follow‐up periods and thus are not accurately captured within our data sets. Finally, screening rates for many of the birth country groups examined had low statistical precision due to the small numbers of women representing these groups within the study cohort.

Despite our findings of lower screening rates among some immigrant populations in BC, it is important to also reflect on the population statistics that our study presented, principally that only 50% of the eligible women participated in breast screening over the 2‐year follow‐up. Further, the 30‐month retention rate for first time attendees in the present study was 44%. These statistics are far below targets set by Canadian cancer screening expert advisory panels.^1^ Some of the observed screening disparities among immigrant sub‐populations will require specific screening promotion to improve screening rates; there are strong equity rationales for such interventions. However, given the relatively small sizes of the immigrant populations within our cohort, raising the screening rates in the immigrant populations alone will not substantially improve the overall population screening rate.

Addressing the observed screening disparities will be a complex task given the diversity of the BC immigrant population. The present paper was not intended as a comprehensive review of potential interventions to address disparities in breast screening; however, the literature related to screening barriers and interventions among immigrants is rich. Our findings highlight that screening rates are generally lowest among new or most recent immigrants suggesting this population as an important focus for intervention. However, designing interventions will be challenging given that, as shown in our findings, recent immigrants to BC are diverse with respect to characteristics such as language, PCP contact, age, and education level. This variation exists even within groups of women that immigrate from a common world region (eg, the Canadian language proficiency, education level and PCP contact among Chinese and Filipino immigrant women differed significantly). Despite this, as a substantial proportion of un‐screened recent immigrants have had PCP contact, interventions mediated through PCPs may be an approach to reach many women. In Canada, recently published studies on interventions to improve screening rates mediated through PCP's reported positive results.^36^, ^37^ Thus if interventions mediated through PCP practices are to be contemplated within BC, further research to better understand how recent immigrant women access primary care and a better characterization of the PCPs they visit would be instrumental to suggest potential interventions.

## CONFLICT OF INTEREST

None declared.
