# Supplementary material for: Breast screening participation and retention among immigrants and nonimmigrants in British Columbia: A population‐based study
Source: Cancer Med. 2018 Jul 9;7(8):4044–67. doi: 10.1002/cam4.1608 (PMC6089191; doi:10.1002/cam4.1608)
Supplement: Supplementary file 1 [file CAM4-7-4044-s001.docx]

**Supplemental Tables**

Table A1: Age-standardized participation rates by country of birth for all countries with 100 or more eligible women in the participation cohort

| **World Region** | **Country** | **Number of Eligible Women** | **Participation Rate [95% CI]** |
| --- | --- | --- | --- |
| Non-immigrant | Non-immigrant | 451881 | 51.2 [51.0,51.3] |
| East Asia/Pacific | Regional Rate | 51378 | 45.4 [44.9,45.8] |
|  | CMHT | 30185 | 45.9 [45.3,46.5] |
|  | Philippines | 10911 | 45.0 [43.9,46.0] |
|  | South Korea | 4028 | 40.1 [38.4,41.7] |
|  | Vietnam | 2089 | 44.9 [42.5,47.2] |
|  | Fiji | 1018 | 42.6 [39.5,45.6] |
|  | Japan | 770 | 41.1 [37.3,44.8] |
|  | Malaysia | 745 | 53.2 [49.6,56.8] |
|  | Singapore | 518 | 46.9 [42.3,51.5] |
|  | Indonesia | 292 | 51.5 [45.6,57.3] |
|  | Thailand | 277 | 42.6 [35.2,50.0] |
|  | Cambodia | 198 | 37.3 [30.3,44.3] |
|  | Brunei | 154 | 59.6 [51.5,67.7] |
|  | Myanmar (Burma) | 113 | 49.5 [40.3,58.6] |
|  | Other Regional Country | 80 | 45.6 [32.6,58.6] |
| South Asia | Regional Rate | 11043 | 45.1 [44.1,46.0] |
|  | India | 9958 | 45.6 [44.6,46.7] |
|  | Pakistan | 584 | 36.1 [32.1,40.2] |
|  | Sri Lanka | 347 | 47.5 [41.9,53.0] |
|  | Bangladesh | 123 | 41.2 [32.4,50.0] |
|  | Other Regional Country | 31 | 36.9 [21.8,51.9] |
| Caribbean/Latin America | Regional Rate | 2946 | 52.6 [50.6,54.5] |
|  | El Salvador | 567 | 53.9 [49.6,58.2] |
|  | Mexico | 478 | 48.2 [42.9,53.4] |
|  | Colombia | 278 | 56.6 [50.4,62.8] |
|  | Peru | 212 | 57.4 [50.4,64.4] |
|  | Guatemala | 177 | 43.5 [35.6,51.3] |
|  | Brazil | 173 | 59.0 [51.3,66.7] |
|  | Nicaragua | 161 | 54.7 [46.8,62.5] |
|  | Chile | 118 | 50.1 [40.5,59.7] |
|  | Trinidad and Tobago | 112 | 47.3 [38.0,56.6] |
|  | Jamaica | 105 | 46.7 [37.0,56.4] |
|  | Argentina | 100 | 51.2 [40.9,61.6] |
|  | Other Regional Country | 465 | 54.3 [49.4,59.2] |
| Middle East/North Africa | Regional Rate | 4261 | 52.2 [50.7,53.8] |
|  | Iran | 3517 | 54.0 [52.3,55.7] |
|  | Iraq | 249 | 44.1 [37.7,50.5] |
|  | Egypt | 110 | 53.7 [45.6,61.9] |
|  | Lebanon | 101 | 42.6 [32.5,52.7] |
|  | Other Regional Country | 284 | 42.6 [36.3,48.9] |
| Eastern Europe/Central Asia | Regional Rate | 6316 | 37.9 [36.6,39.2] |
|  | Former USSR State | 1774 | 35.0 [32.5,37.5] |
|  | Poland | 1442 | 37.8 [35.1,40.5] |
|  | Former Yugoslavia | 1185 | 39.4 [36.4,42.4] |
|  | Romania | 650 | 36.3 [32.2,40.4] |
|  | Czech/Slovak Republics | 425 | 38.3 [33.5,43.1] |
|  | Afghanistan | 367 | 49.0 [43.6,54.3] |
|  | Hungary | 210 | 35.7 [29.3,42.1] |
|  | Bulgaria | 138 | 35.0 [26.0,43.9] |
|  | Other Regional Country | 125 | 42.2 [32.1,52.2] |
| Australia/NZ/US | Regional Rate | 2958 | 47.1 [45.2,48.9] |
|  | United States of America | 2572 | 46.2 [44.2,48.1] |
|  | Australia | 234 | 59.0 [51.6,66.3] |
|  | New Zealand | 147 | 49.2 [40.1,58.2] |
|  | Other Regional Country | 5 | 0.0 [0.0,0.0] |
| Sub-Saharan Africa | Regional Rate | 2088 | 51.3 [49.0,53.6] |
|  | South Africa | 1012 | 55.5 [52.2,58.7] |
|  | Kenya | 248 | 56.2 [49.9,62.6] |
|  | Zimbabwe | 149 | 50.6 [39.8,61.3] |
|  | Tanzania | 124 | 57.8 [49.1,66.4] |
|  | Ethiopia | 118 | 34.7 [24.9,44.6] |
|  | Other Regional Country | 437 | 42.3 [37.1,47.4] |
| Western Europe | Regional Rate | 4912 | 51.2 [49.7,52.7] |
|  | United Kingdom | 2692 | 55.3 [53.3,57.3] |
|  | Germany | 986 | 41.2 [37.9,44.6] |
|  | Switzerland | 248 | 42.2 [35.7,48.8] |
|  | Netherlands | 206 | 55.8 [48.6,63.0] |
|  | Ireland | 169 | 59.6 [51.6,67.6] |
|  | France | 130 | 47.4 [37.9,56.9] |
|  | Other Regional Country | 481 | 50.7 [46.0,55.4] |
| CI = Confidence interval; NZ = New Zealand; US = United States of America; USSR = Union of Soviet Socialist Republics; ; CMHT = China, Macau, Hong Kong, Taiwan | | | |

Table A2: Participation rates for screening eligible women by study factors – grouped by world region of birth for immigrant women

| **Variable** | **Subgroup** | **Population (N=537,783)** | **Non-immigrant (N=451,881)** | **East Asia/ Pacific (N=51,378)** | **South Asia (N=11,043)** | **Caribbean/ Latin America (N=2,946)** | **Middle East/ North Africa (N=4,261)** | **Eastern Europe/ Central Asia (N=6,316)** | **Australia/ NZ/ US (N=2,958)** | **Sub-Saharan Africa (N=2,088)** | **Western Europe (N=4,912)** |
| --- | --- | --- | --- | --- | --- | --- | --- | --- | --- | --- | --- |
| All women | All women | 50.3  [50.2, 50.4] | 51.2  [51.0, 51.3] | 45.3  [44.9, 45.7] | 44.2  [43.3, 45.1] | 51.7  [49.8, 53.5] | 52.0  [50.5, 53.5] | 37.8  [36.6, 39.0] | 47.0  [45.2, 48.8] | 50.9  [48.7, 53.1] | 50.8  [49.4, 52.2] |
| Age | 50-59 | 48.0  [47.8, 48.2] | 48.5  [48.3, 48.7] | 45.1  [44.6, 45.7] | 47.6  [46.3, 49.0] | 50.6  [48.5, 52.7] | 51.9  [50.1, 53.7] | 37.7  [36.4, 39.1] | 46.7  [44.5, 48.9] | 50.3  [47.8, 52.8] | 50.1  [48.5, 51.7] |
|  | 60-69 | 53.9  [53.7, 54.2] | 55.1  [54.9, 55.4] | 45.7  [44.9, 46.6] | 40.8  [39.5, 42.1] | 54.7  [51.0, 58.3] | 52.3  [49.5, 55.2] | 38.1  [35.6, 40.6] | 47.7  [44.4, 51.0] | 52.7  [48.4, 57.1] | 52.9  [50.0, 55.7] |
| Urban/Rural residence | Urban | 51.2  [51.0, 51.3] | 52.3  [52.2, 52.5] | 45.4  [44.9, 45.8] | 44.3  [43.4, 45.3] | 51.7  [49.9, 53.6] | 52.0  [50.5, 53.5] | 38.1  [36.9, 39.3] | 49.2  [47.1, 51.2] | 50.9  [48.7, 53.1] | 52.4  [50.8, 53.9] |
|  | Rural | 45.2  [44.8, 45.5] | 45.3  [45.0, 45.7] | 40.0  [35.6, 44.4] | 36.4  [29.2, 44.1] | 50.0  [39.6, 60.4] | 53.8  [33.4, 73.4] | 29.4  [23.1, 36.3] | 39.4  [35.6, 43.2] | 51.1  [40.3, 61.8] | 44.4  [41.2, 47.6] |
| Income quintile | 1 (lowest) | 43.0  [42.7, 43.3] | 42.9  [42.5, 43.2] | 43.4  [42.6, 44.2] | 42.9  [41.1, 44.7] | 51.7  [48.1, 55.3] | 45.9  [42.1, 49.6] | 35.9  [33.6, 38.3] | 43.8  [39.0, 48.7] | 44.0  [38.9, 49.2] | 46.5  [42.6, 50.5] |
|  | 2 | 48.3  [48.0, 48.6] | 48.8  [48.5, 49.2] | 47.1  [46.2, 48.0] | 44.5  [42.9, 46.1] | 50.2  [46.4, 54.1] | 50.2  [46.8, 53.6] | 35.4  [32.7, 38.1] | 44.1  [39.6, 48.7] | 46.9  [41.5, 52.2] | 48.9  [45.2, 52.5] |
|  | 3 | 51.1  [50.8, 51.4] | 51.9  [51.6, 52.2] | 47.2  [46.3, 48.2] | 45.2  [43.2, 47.3] | 53.4  [49.4, 57.4] | 55.9  [51.7, 59.9] | 39.4  [36.6, 42.1] | 44.4  [40.2, 48.8] | 48.9  [43.8, 53.9] | 49.8  [46.6, 53.0] |
|  | 4 | 52.7  [52.4, 53.0] | 53.5  [53.2, 53.8] | 45.5  [44.4, 46.6] | 42.7  [39.8, 45.5] | 51.5  [47.0, 56.0] | 53.1  [49.7, 56.5] | 40.5  [37.7, 43.5] | 47.3  [43.2, 51.4] | 53.9  [49.0, 58.7] | 51.0  [48.0, 54.0] |
|  | 5 (highest) | 55.7  [55.4, 56.0] | 56.8  [56.5, 57.1] | 43.4  [42.2, 44.6] | 47.1  [43.7, 50.6] | 52.3  [47.1, 57.4] | 55.0  [52.1, 57.9] | 39.3  [36.2, 42.6] | 51.5  [48.2, 54.8] | 57.7  [53.4, 61.9] | 54.7  [52.0, 57.3] |
| # Major ADG's | 0 | 48.3  [48.1, 48.4] | 49.6  [49.4, 49.8] | 41.3  [40.8, 41.8] | 40.9  [39.7, 42.1] | 48.1  [45.7, 50.6] | 47.7  [45.7, 49.6] | 35.1  [33.6, 36.6] | 44.8  [42.5, 47.1] | 48.2  [45.4, 51.0] | 49.0  [47.2, 50.7] |
|  | 1 | 54.4  [54.1, 54.6] | 54.6  [54.3, 54.9] | 55.0  [54.0, 56.0] | 47.8  [46.0, 49.6] | 56.4  [52.8, 59.9] | 57.4  [54.3, 60.3] | 42.0  [39.6, 44.5] | 52.0  [48.2, 55.8] | 55.7  [51.4, 60.0] | 55.0  [52.0, 57.9] |
|  | 2 | 53.6  [53.1, 54.0] | 53.3  [52.9, 53.8] | 57.6  [55.8, 59.4] | 50.2  [47.2, 53.2] | 60.7  [54.8, 66.3] | 59.4  [54.3, 64.3] | 46.2  [41.6, 50.9] | 50.4  [43.9, 57.0] | 52.7  [44.9, 60.4] | 56.3  [50.3, 62.1] |
|  | 3+ | 47.8  [47.2, 48.5] | 47.2  [46.5, 47.9] | 56.3  [53.0, 59.5] | 51.7  [46.6, 56.7] | 50.5  [40.3, 60.7] | 65.6  [56.7, 73.8] | 44.8  [37.5, 52.3] | 50.0  [39.0, 61.0] | 49.1  [35.4, 62.9] | 47.7  [37.9, 57.5] |
|  | Unknown | 50.7  [49.9, 51.4] | 51.7  [50.9, 52.5] | 44.6  [42.2, 47.1] | 44.8  [39.5, 50.3] | 50.5  [40.2, 60.8] | 57.1  [48.0, 65.9] | 32.8  [26.2, 39.9] | 41.9  [32.3, 51.9] | 62.7  [49.1, 75.0] | 52.1  [43.6, 60.4] |
| # PCP visits | 0 | 14.3  [13.9, 14.7] | 16.4  [16.0, 16.9] | 6.7  [ 6.1, 7.4] | 8.2  [ 5.5, 11.6] | 17.2  [11.1, 24.9] | 7.5  [ 4.9, 11.0] | 10.2  [ 7.5, 13.4] | 13.7  [ 9.3, 19.1] | 14.5  [ 8.5, 22.5] | 15.7  [12.3, 19.7] |
|  | 1-4 | 43.1  [42.8, 43.4] | 44.6  [44.3, 44.9] | 33.5  [32.6, 34.4] | 27.4  [24.6, 30.4] | 39.4  [34.7, 44.1] | 38.7  [34.5, 43.1] | 28.5  [26.1, 31.0] | 39.5  [35.9, 43.1] | 41.7  [37.1, 46.3] | 42.6  [39.9, 45.4] |
|  | 5-9 | 54.2  [54.0, 54.5] | 55.1  [54.9, 55.4] | 49.7  [48.8, 50.5] | 40.4  [38.4, 42.5] | 49.3  [45.7, 53.0] | 52.1  [48.8, 55.3] | 38.1  [35.8, 40.4] | 53.4  [50.1, 56.6] | 53.0  [49.0, 56.9] | 56.5  [53.9, 59.0] |
|  | 10-14 | 57.0  [56.7, 57.3] | 57.5  [57.2, 57.8] | 56.7  [55.7, 57.6] | 45.5  [43.5, 47.5] | 55.8  [51.9, 59.7] | 58.7  [55.4, 61.9] | 46.1  [43.3, 48.8] | 55.6  [51.4, 59.7] | 56.7  [51.9, 61.5] | 60.5  [57.3, 63.7] |
|  | 15+ | 55.0  [54.8, 55.3] | 54.4  [54.1, 54.7] | 62.7  [61.7, 63.6] | 50.7  [49.4, 52.1] | 60.8  [57.6, 63.9] | 61.5  [59.1, 63.9] | 46.6  [44.0, 49.1] | 50.2  [45.8, 54.6] | 60.3  [55.8, 64.8] | 58.9  [55.4, 62.3] |
| Prior screening | Yes | 65.1  [65.0, 65.3] | 65.7  [65.5, 65.8] | 61.2  [60.7, 61.7] | 63.7  [62.5, 64.8] | 64.8  [62.7, 66.7] | 64.5  [62.8, 66.2] | 54.5  [52.9, 56.0] | 63.9  [61.8, 66.0] | 67.5  [65.0, 69.9] | 67.3  [65.7, 68.8] |
| Years of residence in Canada † | < 5 | 37.0  [35.8, 38.2] | NA | 34.1  [32.4, 35.9] | 34.9  [32.6, 37.3] | 48.2  [41.1, 55.4] | 50.6  [45.6, 55.7] | 27.1  [21.6, 33.3] | 42.9  [37.3, 48.5] | 38.4  [30.4, 46.8] | 48.8  [43.4, 54.3] |
|  | 5 - 9 | 39.3  [38.5, 40.2] |  | 36.7  [35.5, 37.9] | 38.0  [36.2, 39.9] | 48.6  [43.6, 53.7] | 52.6  [49.0, 56.2] | 37.6  [34.0, 41.4] | 42.9  [39.0, 46.9] | 42.2  [35.7, 49.0] | 47.8  [43.7, 51.9] |
|  | 10 - 19 | 45.9  [45.4, 46.3] |  | 45.2  [44.6, 45.7] | 48.3  [46.9, 49.6] | 50.4  [47.0, 53.8] | 52.1  [49.9, 54.2] | 38.1  [36.4, 39.8] | 47.8  [44.3, 51.2] | 51.5  [48.3, 54.7] | 50.2  [47.8, 52.7] |
|  | 20+ | 50.6  [50.0, 51.2] |  | 51.5  [50.7, 52.3] | 51.4  [48.8, 54.0] | 53.6  [51.1, 56.1] | 51.9  [48.7, 55.1] | 38.6  [36.6, 40.7] | 49.6  [46.8, 52.5] | 55.2  [51.6, 58.8] | 52.2  [50.2, 54.3] |

PCP = Primary care physician; ADG = aggregate diagnosis group; NA = Not applicable; NZ = New Zealand; US = United States of America

† Years of residence in Canada for the “Population” column refers to the pooled group of all immigrants

Table A3: Age-standardized retention rates by country of birth for all countries with 100 or more eligible women in the retention cohort

|  | | | | **Retention Rate [95% CI]** | |
| --- | --- | --- | --- | --- | --- |
| **World Region** | **Country** | **Number of Eligible Women** | **% First Screen** | **All Women** | **Prior Screening** |
| Non-immigrant | Non-immigrant | 245123 | 5.6 | 74.4 [74.2,74.5] | 76.2 [76.0,76.4] |
| East Asia/Pacific | Regional Rate | 21369 | 8.1 | 73.3 [72.7,74.0] | 75.4 [74.8,76.1] |
|  | CMHT | 12863 | 6.3 | 74.3 [73.5,75.1] | 76.1 [75.3,76.9] |
|  | Philippines | 4324 | 11.4 | 71.4 [69.8,73.1] | 73.7 [72.0,75.4] |
|  | South Korea | 1553 | 11.3 | 66.3 [63.8,68.8] | 68.8 [66.2,71.4] |
|  | Vietnam | 849 | 9.5 | 74.4 [71.1,77.7] | 77.4 [74.0,80.7] |
|  | Fiji | 439 | 13.9 | 67.3 [62.9,71.7] | 72.0 [67.5,76.6] |
|  | Malaysia | 379 | 6.3 | 77.9 [73.6,82.1] | 80.5 [76.3,84.7] |
|  | Japan | 272 | 11.0 | 73.8 [67.7,79.9] | 76.5 [70.2,82.9] |
|  | Singapore | 224 | 5.4 | 77.2 [71.3,83.1] | 79.7 [74.4,85.1] |
|  | Indonesia | 142 | 6.3 | 77.4 [70.9,83.8] | 79.1 [72.6,85.6] |
|  | Other Regional Country | 324 | 10.8 | 76.9 [71.9,82.0] | 79.8 [74.8,84.8] |
| South Asia | Regional Rate | 4470 | 22.3 | 69.5 [68.1,70.8] | 75.5 [74.0,76.9] |
|  | India | 4054 | 22.7 | 69.9 [68.4,71.3] | 76.1 [74.6,77.6] |
|  | Pakistan | 209 | 19.1 | 67.9 [61.3,74.4] | 74.7 [68.5,80.8] |
|  | Sri Lanka | 152 | 18.4 | 62.3 [54.0,70.6] | 65.5 [56.5,74.5] |
|  | Other Regional Country | 55 | 16.4 | 67.2 [53.5,80.8] | 73.4 [58.7,88.2] |
| Caribbean/Latin America | Regional Rate | 1385 | 9.5 | 70.8 [68.2,73.4] | 73.4 [70.8,76.1] |
|  | El Salvador | 300 | 7.0 | 73.1 [67.7,78.5] | 74.4 [68.9,79.9] |
|  | Mexico | 193 | 10.4 | 67.6 [60.0,75.2] | 70.3 [62.4,78.2] |
|  | Colombia | 121 | 11.6 | 71.5 [62.1,81.0] | 69.8 [59.6,79.9] |
|  | Peru | 109 | 9.2 | 75.5 [67.7,83.4] | 80.6 [73.5,87.7] |
|  | Other Regional Country | 662 | 10.1 | 69.3 [65.6,73.0] | 72.3 [68.4,76.1] |
| Middle East/North Africa | Regional Rate | 1985 | 11.7 | 70.3 [68.1,72.4] | 72.5 [70.3,74.8] |
|  | Iran | 1716 | 11.4 | 70.3 [67.9,72.6] | 72.6 [70.2,75.0] |
|  | Other Regional Country | 269 | 13.4 | 70.0 [64.3,75.7] | 71.9 [65.9,77.9] |
| Eastern Europe/Central Asia | Regional Rate | 2230 | 11.9 | 68.6 [66.5,70.8] | 72.2 [70.0,74.4] |
|  | Former USSR State | 539 | 16.0 | 67.7 [63.1,72.2] | 73.1 [68.5,77.7] |
|  | Poland | 534 | 10.9 | 68.9 [64.7,73.1] | 72.0 [67.7,76.3] |
|  | Former Yugoslavia | 440 | 9.8 | 70.8 [65.8,75.7] | 73.8 [68.9,78.8] |
|  | Romania | 222 | 9.9 | 61.6 [54.8,68.4] | 65.1 [58.3,71.8] |
|  | Czech/Slovak Republics | 174 | 5.7 | 72.3 [65.6,78.9] | 75.1 [68.4,81.7] |
|  | Afghanistan | 161 | 18.0 | 67.7 [60.1,75.4] | 71.1 [62.7,79.5] |
|  | Other Regional Country | 160 | 10.6 | 74.6 [67.7,81.6] | 75.2 [68.0,82.4] |
| Australia/NZ/US | Regional Rate | 1305 | 9.5 | 68.8 [66.2,71.4] | 71.6 [68.9,74.3] |
|  | United States of America | 1141 | 9.8 | 68.9 [66.1,71.6] | 71.8 [69.0,74.6] |
|  | Other Regional Country | 164 | 7.3 | 68.2 [59.6,76.7] | 69.3 [60.5,78.1] |
| Sub-Saharan Africa | Regional Rate | 968 | 9.6 | 75.2 [72.3,78.1] | 77.0 [74.0,79.9] |
|  | South Africa | 501 | 7.2 | 76.1 [72.0,80.2] | 77.1 [72.9,81.3] |
|  | Kenya | 135 | 8.1 | 77.8 [70.9,84.8] | 79.9 [72.8,87.0] |
|  | Other Regional Country | 332 | 13.9 | 73.1 [67.9,78.3] | 75.6 [70.3,80.9] |
| Western Europe | Regional Rate | 2217 | 8.4 | 76.5 [74.6,78.3] | 77.9 [76.0,79.8] |
|  | United Kingdom | 1318 | 7.8 | 78.0 [75.7,80.4] | 79.3 [76.9,81.7] |
|  | Germany | 359 | 12.3 | 73.0 [68.1,77.8] | 74.9 [69.9,79.9] |
|  | Other Regional Country | 540 | 7.2 | 74.5 [70.5,78.4] | 75.9 [71.9,79.9] |
| CI = Confidence interval; NZ = New Zealand; US = United States of America; USSR = Union of Soviet Socialist Republics; CMHT = China, Macau, Hong Kong, Taiwan | | | | | |
